# Guessing with a Bit of Help †

**DOI:** 10.3390/e22010039

**Published:** 2019-12-26

**Authors:** Nir Weinberger, Ofer Shayevitz

**Affiliations:** 1 Institute for Data, Systems, and Society and Laboratory for Information & Decision Systems, Massachusetts Institute of Technology, Cambridge, MA 02139, USA; 2 Department of Electrical Engineering-Systems, Tel Aviv University, Tel Aviv 69978, Israel; ofersha@eng.tau.ac.il

**Keywords:** boolean functions, fourier analysis, guessing moments, guessing with a helper, hypercontractivity, maximum entropy, strong data-processing inequalities

## Abstract

What is the value of just a few bits to a guesser? We study this problem in a setup where Alice wishes to guess an independent and identically distributed (i.i.d.) random vector and can procure a fixed number of *k* information bits from Bob, who has observed this vector through a memoryless channel. We are interested in the *guessing ratio*, which we define as the ratio of Alice’s guessing-moments with and without observing Bob’s bits. For the case of a uniform binary vector observed through a binary symmetric channel, we provide two upper bounds on the guessing ratio by analyzing the performance of the dictator (for general k≥1) and majority functions (for k=1). We further provide a lower bound via maximum entropy (for general k≥1) and a lower bound based on Fourier-analytic/hypercontractivity arguments (for k=1). We then extend our maximum entropy argument to give a lower bound on the guessing ratio for a general channel with a binary uniform input that is expressed using the strong data-processing inequality constant of the reverse channel. We compute this bound for the binary erasure channel and conjecture that greedy dictator functions achieve the optimal guessing ratio.

## 1. Introduction

In the classical guessing problem, Alice wishes to learn the value of a discrete random variable (r.v.) *X* as quickly as possible by sequentially asking yes/no questions of the form “Is X=x?”, until she makes a correct guess. A guessing strategy corresponds to an ordering of the alphabet of *X* according to which the guesses are made and induces a random guessing time. It is well known and simple to verify that the guessing strategy which simultaneously minimizes all the positive moments of the guessing time is to order the alphabet according to a decreasing order of probability. Formally, for any s>0, the *minimal sth-order guessing-time moment* of *X* is

(1) $$G_{s}\left(X\right):=E\left({\mathrm{ORD}}_{X}^{s}\left(X\right)\right),$$

where ${\mathrm{ORD}}_{X}\left(x\right)$ returns the index of the symbol *x* relative to the order induced by sorting the probabilities in a descending order, with ties broken arbitrarily. For brevity, we refer to $G_{s}\left(X\right)$ as the *guessing-moment* of *X*.

Several motivating problems for studying guesswork are fairness in betting games, computational complexity of sequential decoding [1], computational complexity of lossy source coding and database search algorithms (see the introduction of Reference [2] for a discussion), secrecy systems [3,4,5], and crypt-analysis (password cracking) [6,7]. The guessing problem was first introduced and studied in an information-theoretic framework by Massey [8], who drew a relation between the average guessing time of an r.v. to its entropy. It was later explored more systematically by Arikan [1], who also introduced the problem of guessing with side information. In this problem, Alice is in possession of another r.v. *Y* that is jointly distributed with *X*, and then, the optimal conditional guessing strategy is to guess by decreasing order of conditional probabilities. Hence, the associated *minimal conditional sth-order guessing-time moment of X given Y* is

(2) $$G_{s}\left(X|Y\right):=E\left({\mathrm{ORD}}_{X|Y}^{s}\left(X|Y\right)\right),$$

where ${\mathrm{ORD}}_{X|Y}\left(x|y\right)$ returns the index of *x* relative to the order induced by sorting the conditional probabilities of *X* given that Y=y in a descending order. Arikan showed that, as intuition suggests, side information reduces the guessing-moments ([1], Corollary 1)

(3) $$G_{s}\left(X|Y\right)\leq G_{s}\left(X\right).$$

Furthermore, he showed that, if ${\left\{\left(X_{i},Y_{i}\right)\right\}}_{i=1}^{n}$ is an i.i.d. sequence, then ([1], Proposition 5)

(4) $$\lim_{n\to \infty }\frac{1}{n}\log G_{s}^{1/s}\left(X^{n}|Y^{n}\right)=H_{\frac{1}{1+s}}\left(X_{1}|Y_{1}\right),$$

where $H_{\alpha }\left(X|Y\right)$ is the Arimoto-Rényi conditional entropy of order α. As was noted by Arikan a few years later [9], the guessing moments are related to the large deviations behavior of the random variable $\frac{1}{n}\log{\mathrm{ORD}}_{X^{n}|Y^{n}}\left(X^{n}|Y^{n}\right)$. However, in Reference [9], he was only able to obtain right-tail large deviation bounds since asymptotically tight bounds on $G_{s}\left(X^{n}|Y^{n}\right)$ were only known for positive moments (s>0). Large deviation principle for the normalized logarithm of the guessing time was later established in Reference [10] using substantial results from References [11,12]. Throughout the years, information-theoretic analysis of the guessing problem was extended in multiple directions, such as guessing until the distortion between the guess and the true value is below a certain threshold [2], guessing under source uncertainty [13], and improved bounds at finite blocklength [14,15,16], to name a few.

In the conditional setting described above, one may think of $Y^{n}$ as side information observed by a “helper”, say Bob, who sends his observations to Alice. Nonetheless, as other problems employing a helper (e.g., source coding [17,18]), it is more realistic to impose communication constraints and to assume that Bob can only send a compressed description of $Y^{n}$ to Alice. This setting was recently addressed by Graczyk and Lapidoth [19,20], who considered the case where Bob encodes $Y^{n}$ at a positive rate using nR bits before sending this description to Alice. They then characterized the best possible guessing-moments attained by Alice for general distributions as a function of the rate *R*. In this paper, we take this setting to its extreme and attempt to quantify the value of *k bits* in terms of reducing the guessing-moments by allowing Bob to use only a *k*-bit description of $Y^{n}$. The major difference from previous work is that, here, *k* is finite and does not increase with *n*, and for some of our results, we further concentrate on the extreme case of k=1—a single bit of help. To that end, we define (Section 2) the *guessing ratio*, which is the (asymptotically) best possible ratio of the guessing-moments of $X^{n}$ obtained with and without observing a function $f\left(Y^{n}\right)\in {\left\{0,1\right\}}^{k}$, i.e., the minimal possible ratio $G_{s}\left(X^{n}|f\left(Y^{n}\right)\right)/G_{s}\left(X^{n}\right)$ as a function of s>0, in the limit of large *n*.

Sharply characterizing the guessing ratio appears to be a difficult problem in general. Here, we mostly focus on the special case where $X^{n}$ is uniformly distributed over the Boolean cube ${\left\{0,1\right\}}^{n}$ and $Y^{n}$ is obtained by passing $X^{n}$ through a memoryless binary symmetric channel (BSC) with crossover probability δ (Section 3). We derive two upper bounds and two lower bounds on the guessing ratio in this case. The upper bounds are derived by analyzing the ratio attained by two specific functions, *k*-*Dictator*, to wit $f\left(Y^{n}\right)=Y^{k}$, and *Majority*, to wit $f\left(Y^{n}\right)=𝟙\left(\sum_{i=1}^{n}Y_{i}>\frac{n}{2}\right)$, where 𝟙(·) is the indicator function, and for simplicity, we henceforth assume that *n* is odd when discussing majority functions. For k=1, we demonstrate that neither of these functions is better than the other for all values of the moment order *s*. The first lower bound is based on relating the guessing-moment to entropy using maximum-entropy arguments (generalizing a result of Reference [8]), and the second one on Fourier-analytic techniques combined with a hypercontractivity argument [21]. Furthermore, for the restricted class of functions for which the constituent *k*-bit functions operate on disjoint sets of bits, a general method is proposed for transforming a lower bound valid for k=1 to a lower bound valid for any k≥1. Nonetheless, we remark that our bounds are valid for s>0 and obtaining similar bounds for s<0 in order to obtain large deviation principle for the normalized logarithm of the guessing time remains an open problem. In Section 4, we briefly discuss the more general case where $X^{n}$ is still uniform over the Boolean cube, but $Y^{n}$ is obtained from $X^{n}$ via a general binary-input, arbitrary-output channel. We generalize our entropy lower bound to this case using the strong data-processing inequality (SDPI) applied to the reverse channel (from *Y* to *X*). We then discuss the case of the binary erasure channel (BEC), for which we also provide an upper bound by analyzing the *greedy dictator* function, namely where Bob sends the first bit that has not been erased. We conjecture that this function minimizes the guessing-moments simultaneously at all erasure parameters and all moments *s*.

*Related Work.* As mentioned above, Graczyk and Lapidoth [19,20] considered the same guessing question if Bob can communicate with Alice at some positive rate *R*, i.e., can use k=nR bits to describe $Y^{n}$. This setup facilitates the use of large-deviation-based information-theoretic techniques, which allowed the authors to characterize the optimal reduction in the guessing-moments as a function of *R* to the first order in the exponent. This type of argument cannot be applied in our setup of finite number of bits. Furthermore, as we shall see, in our setup, the exponential order of the guessing moment with help is equal to the one without it and the performance is therefore more finely characterized by bounding the ratio of the guessing-moments. For a single bit of help k=1, characterizing the guessing ratio in the case of the BSC with a uniform input can also be thought of as a guessing variant of the *most informative Boolean function problem* introduced by Kumar and Courtade [22]. There, the maximal reduction in the entropy of $X^{n}$ obtainable by observing a Boolean function $f(Y^{n})$ is sought after. It was conjectured in Reference [22] that a *dictator function*, e.g., $f\left(y^{n}\right)=y_{1}$, is optimal simultaneously at all noise levels; see References [23,24,25,26] for some recent progress. As in the guessing case, allowing Bob to describe $Y^{n}$ using nR bits renders the problem amenable to an exact information-theoretic characterization [27]. In another related work [28], we have asked about the Boolean function $Y^{n}$ that maximizes the reduction in the sequential mean-squared prediction error of $X^{n}$ and showed that the majority function is optimal in the noiseless case. There is, however, no single function that is simultaneously optimal at all noise levels. Finally, in a recent line of works [29,30], the average guessing time using the help of a noisy version of $f(X^{n})$ has been considered. The model in this paper is different since the noise is applied to the inputs of the function rather than to its output.

## 2. Problem Statement

Let $X^{n}$ be an i.i.d. vector from a distribution $P_{X}$, which is transmitted over a memoryless channel of conditional distribution $P_{Y|X}$. A helper observes $Y^{n}\in Y^{n}$ at the output of the channel and can send *k* bits $f\left(Y^{n}\right),\;f:Y^{n}\to {\left\{0,1\right\}}^{k}$ to a guesser of $X^{n}$. Our goal is to characterize the best possible multiplicative reduction in guessing-moments offered by a function *f*, in the limit of large *n*. Precisely, we wish to characterize the *guessing ratio*, defined as

(5) $$\gamma_{s,k}\left(P_{X},P_{Y|X}\right):=\underset{n\to \infty }{lim sup}\min_{f:Y^{n}\to {\left\{0,1\right\}}^{k}}\frac{G_{s}\left(X^{n}|f\left(Y^{n}\right)\right)}{G_{s}\left(X^{n}\right)}$$

for an arbitrary s>0. In this paper, we are mostly interested in the case where $P_{X}=\left(1/2,1/2\right)$, i.e., $X^{n}$ is uniformly distributed over ${\left\{0,1\right\}}^{n}$, and where the channel is a BSC with crossover probability δ∈[0,1/2]. With a slight abuse of notation, we denote the guessing ratio in this case by $\gamma_{s,k}\left(\delta \right)$. Furthermore, some of the results will be restricted to the case of a single bit of help (k=1), and in this case, we will further abbreviate the notation from $\gamma_{s,1}\left(\delta \right)$ to $\gamma_{s}\left(\delta \right)$. We note the following basic facts.

**Proposition** **1.** *The following properties hold:*

*1.*  *The minimum in Equation (5) is achieved by a sequence of deterministic functions.*
*2.*  *$\gamma_{s,k}\left(\delta \right)$ is a non-decreasing function of δ∈[0,1/2] which satisfies $\gamma_{s,k}\left(0\right)=2^{-sk}$ and $\gamma_{s,k}\left(1/2\right)=1$. In addition, $\gamma_{s,k}\left(0\right)$ is attained by any sequence of functions $f_{n}$ such that $f_{n}\left(Y^{n}\right)$ is a uniform Bernoulli vector, i.e., $\mathrm{Pr}\left(f_{n}\left(Y^{n}\right)=b^{k}\right)=2^{-k}$ for all $b^{k}\in {\left\{0,1\right\}}^{k}$.*
*3.*  *For a BSC $P_{Y|X}$, the limit-supremum in Equation (5) defining $\gamma_{s,k}\left(\delta \right)$ is a regular limit.*
*4.*  *If k=1 and $X^{n}$ is a uniformly distributed vector, then the optimal guessing order given that $f(Y^{n})=0$ is reversed to the optimal guessing order when $f(Y^{n})=1$.*

**Proof.**  See Appendix A. □

## 3. Guessing Ratio for a Binary Symmetric Channel

### 3.1. Main Results

We begin by presenting the bound on the guessing ratio $\gamma_{s,k}\left(\delta \right)$ obtained by *k*-*dictator* functions and then proceed to the bound obtained by *majority* functions for a single bit of help, k=1. The proofs are given in the next two subsections.

**Theorem** **1.** *Let Lk,w:=∑v=0wkv for w∈{0,1,…,k}. The guessing ratio is upper bounded as*

(6) $$\gamma_{s,k}\left(\delta \right)\leq \left(1-2\delta \right)\cdot 2^{-sk}\cdot \sum_{w=0}^{k-1}{\left(1-\delta \right)}^{k-1-w}\delta^{w}\cdot L_{k,w}^{s+1}+{\left(2\delta \right)}^{k},$$

*and this upper bound is achieved by k-dictator functions, $f\left(y^{n}\right)=y^{k}$.*

Specifically, for k=1, Theorem 1 implies

(7) $$\gamma_{s}\left(\delta \right)\leq \left(1-2\delta \right)\cdot 2^{-s}+2\delta .$$

**Theorem** **2.** *Let $\beta :=\frac{1-2\delta }{\sqrt{4\delta (1-\delta )}}$ and Z∼N(0,1), and denote by Q(·) the tail distribution function of the standard normal distribution. Then, the guessing ratio is upper bounded as*

(8) $$\gamma_{s}\left(\delta \right)\leq 2\cdot \left(s+1\right)\cdot E\left[Q\left(\beta Z\right)\cdot {\left(1-Q(Z)\right)}^{s}\right],$$

*and this upper bound is achieved by majority functions, $f\left(y^{n}\right)=𝟙\left(\sum_{i=1}^{n}y_{i}>\frac{n}{2}\right)$.*

We remark that, if k=1, the guessing ratio of functions similar to the dictator and majority functions, such as single-bit dictator on j>1 inputs ($f(y^{n})=1$ if and only if $y^{j}=1^{j}$) or unbalanced majority ($f\left(y^{n}\right)=𝟙\left(\sum_{i=1}^{n}y_{i}>t\right)$ for some *t*), may also be analyzed in a similar way. However, numerical computations indicate that they do not improve the bounds of Theorems 1 and 2, and thus, their analysis is omitted.

We next present two lower bounds on the guessing ratio $\gamma_{s,k}\left(\delta \right)$. The first is based on maximum-entropy arguments, and the second is based on Fourier-analytic arguments.

**Theorem** **3.** *The guessing ratio satisfies the following lower bound:*

(9) $$\gamma_{s,k}\left(\delta \right)\geq e^{-1}\cdot \frac{s^{s-1}\cdot \left(s+1\right)}{\Gamma^{s}\left(\frac{1}{s}\right)}\cdot 2^{-sk{\left(1-2\delta \right)}^{2}}$$

*where $\Gamma \left(z\right):=\int_{0}^{\infty }t^{z-1}e^{-t}dt$ is Euler’s Gamma function (defined for ℜ{z}>0).*

**Remark** **1.** *When restricted to k=1, the proof of Theorem 3 utilizes the bound $H\left(X^{n}|f\left(Y^{n}\right)\right)\geq n-{\left(1-2\delta \right)}^{2}$ (see Equation (63)). For balanced functions, this bound was improved in Reference [23] for $1/2(1-1/\sqrt{3})\leq \delta \leq 1/2$. Using this improved bound here leads to an immediate improvement in the bound of Theorem 3. Furthermore, it is known [24] that there exists $\delta_{0}$ such that the most informative Boolean function conjecture holds for all $\delta_{0}\leq \delta \leq 1/2$. For such crossover probabilities,*

(10) $$H\left(X^{n}|f\left(Y^{n}\right)\right)\geq n-1+h\left(\delta \right)$$

*holds, and then, Theorem 3 may be improved to*

(11) $$\gamma_{s}\left(\delta \right)\geq e^{-1}\cdot \frac{s^{s-1}\cdot \left(s+1\right)}{\Gamma^{s}\left(\frac{1}{s}\right)}\cdot 2^{-s\left(1-h(\delta )\right)}.$$

Our Fourier-based bound for k=1 is as follows:

**Theorem** **4.** *Let $\tau :=1+{\left(1-2\delta \right)}^{2(1-\lambda )}$. The guessing ratio is lower bounded as*

(12) $$\gamma_{s}\left(\delta \right)\geq \max_{0\leq \lambda \leq 1}\left[1-\frac{\left(s+1\right)\cdot {\left(1-2\delta \right)}^{\lambda }}{{\left(\tau s+1\right)}^{1/\tau }}\right].$$

This bound can be weakened by the possibly suboptimal choice λ=1, which leads to a simpler yet explicit bound:

**Corollary** **1.** (13) $$\gamma_{s}\left(\delta \right)\geq 1-\frac{\left(s+1)\cdot (1-2\delta \right)}{\sqrt{1+2s}}.$$

The bound in Theorem 4 is only valid for the case k=1. An interesting problem is to find a general way of “transforming” a lower bound which assumes k=1 to a bound useful for k>1. In principle, such a result could stem from the observation that a *k* bit function provides *k* different conditional optimal guessing orders for each of its output bits. For a general function, however, distilling a useful bound from this observation seems challenging since the relation between the optimal guessing order induced by each of the bits and the optimal guessing order induced by all *k* bits might be involved. Nonetheless, such a result is possible to obtain if each of the *k* single-bit functions operate on a different set of input bits. For this restricted set of functions, there is a simple bound which relates the optimal ordering given each of the bits and all the *k* bits together. It is reasonable to conjecture that this restricted sub-class is optimal or at least close to optimal, since it seems that more information is transferred to the guesser when the *k* functions operate on different sets of bits, which make the *k* functions statistically independent.

Specifically, let us specify a *k*-bit function $f:Y^{n}\to {\left\{0,1\right\}}^{k}$ by its *k* constituent one-bit functions $f_{j}:Y^{n}\to \left\{0,1\right\}$, j∈[k]. Let $F_{k}$ be the set of sequences of functions $\left\{f^{\left(n\right)}\right\}$, $f^{\left(n\right)}:Y^{n}\to {\left\{0,1\right\}}^{k}$, such that each specific sequence of functions $\left\{f^{\left(n\right)}\right\}$ satisfies the following property: There exists a sequence of partitions ${\left\{{\left\{I_{j}^{\left(n\right)}\right\}}_{j\in [k]}\right\}}_{n=1}^{\infty }$ of [n], such that, for all n≥1 and j∈[k], $f_{j}^{\left(n\right)}\left(Y^{n}\right)$ only depends on ${\left\{Y_{i}\right\}}_{i\in I_{j}^{\left(n\right)}}$ and $\lim_{n\to \infty }\left|I_{j}^{\left(n\right)}\right|=\infty$ for all j∈[k]. In particular, this implies that ${\left\{f_{j}^{\left(n\right)}\left(Y^{n}\right)\right\}}_{j\in [k]}$ is mutually independent for all n≥1. For example, when k=2, $f_{1}\left(x^{n}\right)=x_{1}$, and $f_{2}\left(x^{n}\right)=x_{2}$, we can choose $I_{j}^{\left(n\right)}$ to be the odd/even indices. For $f_{1}=\mathrm{Maj}\left(y_{1}^{n/2}\right)$ and $f_{2}=\mathrm{Maj}\left(y_{n/2+1}^{n}\right)$, the sets are the first and second halves of [n]. As in Equation (5), we may define the guessing ratio of this constrained set of functions as

(14) $${\tilde{\gamma }}_{s,k}\left(\delta \right):=\min_{{\left\{f^{\left(n\right)}\right\}}_{n=1}^{\infty }\in F_{k}}\underset{n\to \infty }{lim sup}\frac{G_{s}\left(X^{n}|f^{\left(n\right)}\left(Y^{n}\right)\right)}{G_{s}\left(X^{n}\right)},$$

where, in general, ${\tilde{\gamma }}_{s,k}\left(\delta \right)\geq \gamma_{s,k}\left(\delta \right)$.

**Proposition** **2.** (15) $${\tilde{\gamma }}_{s,k}\left(\delta \right)\geq \frac{{\tilde{\gamma }}_{s,1}^{k}\left(\delta \right)}{{\left(s+1\right)}^{k-1}}.$$

**Figure 1:**
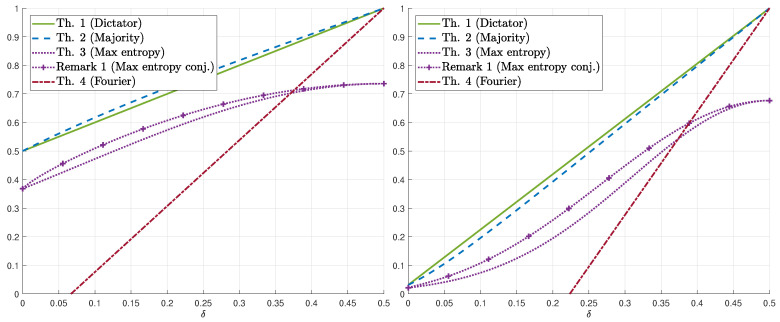
Bounds on $\gamma_{s}\left(\delta \right)$ for s=1 (**left**) and s=5 (**right**) as a function of δ∈[0,1/2].

**Figure 2:**
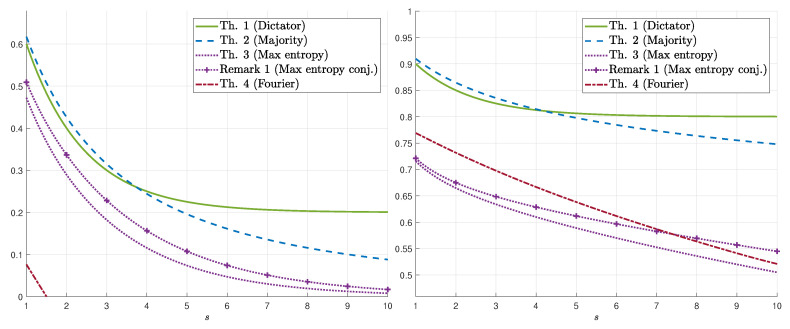
Bounds on $\gamma_{s}\left(\delta \right)$ for δ=0.1 (**left**) and δ=0.4 (**right**) as a function of s∈[1,10].

**Figure 3:**
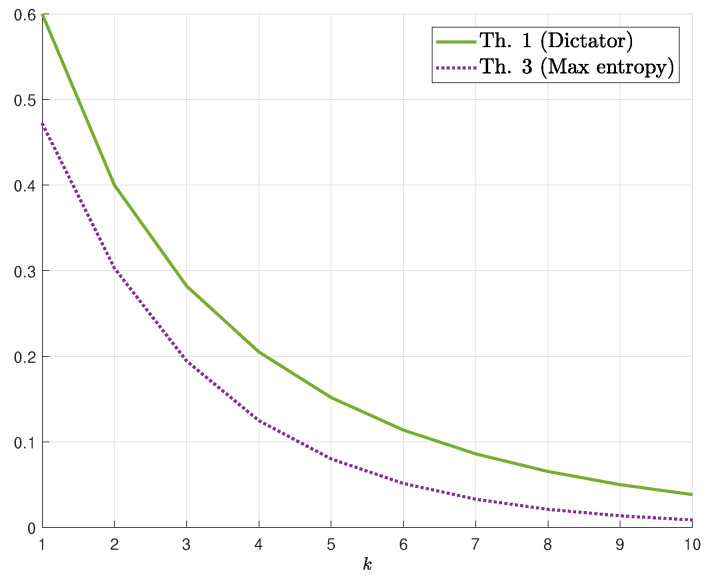
Bounds on $\gamma_{s,k}\left(\delta \right)$ for δ=0.1 and s=1 as a function of *k*.

We demonstrate our results for k=1 in Figure 1 (resp. Figure 2) which display the bounds on $\gamma_{s}\left(\delta \right)$ for fixed values of *s* (resp. δ). The numerical results show that, for the upper bounds, when s≲3.5, dictator dominates majority (for all values of δ), whereas for s≳4.25, majority dominates dictator. For 3.5≲s≲4.25, there exists $\delta_{s}^{\prime }$ such that majority is better for $\delta \in (0,\delta_{s}^{\prime })$ and dictator is better for $\delta \in (\delta_{s}^{\prime },1/2)$. Figure 2 demonstrates the switch from dictator to majority as *s* increases (depending on δ). As for lower bounds, we first remark that the conjectured maximum-entropy bound (Equation (11)) is also plotted (see Remark 1). The numerical results show that the maximum-entropy bound is better for low values of δ whereas the Fourier-analysis bound is better for high values of δ. As a function of *s*, the maximum-entropy bound (resp. Fourier-analysis bound) is better for high (resp. low) values of *s*. We also mention that, in these figures, the maximizing parameter in the Fourier-based bound (Theorem 4) is λ=1 and the resulting bound is as in Equation (13). However, for values of *s* as low as 10, the maximizing λ may be far from 1, and in fact, it continuously and monotonically increases from 0 to 1 as δ increases from 0 to 1/2. Finally, Figure 3 demonstrates the behavior of the *k*-dictator and maximum-entropy bounds on $\gamma_{s,k}\left(\delta \right)$ as a function of *k*.

### 3.2. Proofs of the Upper Bounds on $\gamma_{s,k}\left(\delta \right)$

Let a,b∈N, a≤b be given. The following sum will be useful for the proofs in the rest of the paper:

(16) $$K_{s}\left(a,b\right):=\frac{1}{b-a}\sum_{i=a+1}^{b}i^{s},$$

where we will abbreviate $K_{s}\left(b\right):=K_{s}\left(0,b\right)$. For a pair of sequences ${\left\{a_{n}\right\}}_{n=1}^{\infty }$, {$b_{n}{\}}_{n=1}^{\infty }$, we will let $a_{n}≐b_{n}$ mean that $\lim_{n\to \infty }\frac{a_{n}}{b_{n}}=1$.

**Lemma** **1.** *Let ${\left\{a_{n}\right\}}_{n=1}^{\infty }$ and ${\left\{b_{n}\right\}}_{n=1}^{\infty }$ be non-decreasing integer sequences such that $a_{n}<b_{n}$ for all n and $\lim_{n\to \infty }\left(a_{n}+1\right)/b_{n}=0$. Then, *

(17) $$K_{s}\left(a_{n},b_{n}\right)≐\frac{1}{s+1}\cdot \frac{b_{n}^{s+1}-a_{n}^{s+1}}{b_{n}-a_{n}}.$$

*Specifically, $G_{s}\left(X^{n}\right)=K_{s}\left(2^{n}\right)≐\frac{2^{sn}}{s+1}$.*

**Proof.**  See Appendix A. □

We next prove Theorem 1.

**Proof** **of** **Theorem** **1.** Consider a *k*-dictator function which directly outputs *k* of the bits of $y^{n}$, say, without loss of generality (w.l.o.g.) $f\left(y^{n}\right)=y^{k}$. Let $d_{H}\left(x^{n},y^{n}\right)$ be the Hamming distance of $x^{n}$ and $y^{n}$, and recall the assumption 0<δ<1/2. It is easily verified that the optimal guessing order of $X^{n}$ given $y^{k}$ has k+1 parts, such that the *w*th part, w∈{0,1,…,k}, is comprised of an arbitrary ordering of the kw·2n−k vectors for which $d_{H}\left(x^{k},y^{k}\right)=w$. From symmetry, $G_{s}\left(X^{n}|f\left(Y^{n}\right)\right)=G_{s}\left(X^{n}|f\left(Y^{n}\right)=b^{k}\right)$ for any $b^{k}\in {\left\{0,1\right\}}^{k}$. Then, from Lemma 1

(18) Gs(Xn∣f(Yn)=bk)=∑w=0kkw(1−δ)k−wδw·Ks(2n−k·Lk,w−1,2n−k·Lk,w)

(19) =∑w=0kkw(1−δ)k−wδw·Ks(2n−k·Lk,w−1,2n−k·Lk,w)

(20) ≐∑w=0kkw(1−δ)k−wδw·2s(n−k)s+1·Lk,ws+1−Lk,w−1s+1kw

(21) $$\begin{array}{cc} \; & =\frac{2^{s(n-k)}}{s+1}\sum_{w=0}^{k}{\left(1-\delta \right)}^{k-w}\delta^{w}\cdot \left[L_{k,w}^{s+1}-L_{k,w-1}^{s+1}\right] \end{array}$$

(22) $$\begin{array}{cc} \; & =\frac{2^{s(n-k)}}{s+1}\left(\left(1-2\delta \right)\sum_{w=0}^{k-1}{\left(1-\delta \right)}^{k-1-w}\delta^{w}\cdot L_{k,w}^{s+1}+\delta^{k}2^{k(s+1)}\right) \end{array}$$

where in the first equality, $L_{k,-1}:=0$, and the last equality is obtained by telescoping the sum. The result then follows from Equation (5) and Lemma 1. □

We next prove Theorem 2.

**Proof** **of** **Theorem** **2.** Recall that we assume for simplicity that *n* is odd. The analysis for an even *n* is not fundamentally different. To evaluate the guessing-moment, we first need to find the optimal guessing strategy. To this end, we let $W_{H}\left(x^{n}\right)$ be the Hamming weight of $x^{n}$ and note that the posterior probability is given by

(23) $$\begin{array}{cc} \mathrm{Pr}(X^{n}=x^{n}|\mathrm{Maj}\left(Y^{n}\right)=1) & =\frac{\mathrm{Pr}\left(\mathrm{Maj}\left(Y^{n}\right)=1|X^{n}=x^{n}\right)\cdot \mathrm{Pr}\left(X^{n}=x^{n}\right)}{\mathrm{Pr}(\mathrm{Maj}\left(Y^{n}\right)=1)} \end{array}$$

(24) $$\begin{array}{cc} \; & =2^{1-n}\cdot \mathrm{Pr}\left(\sum_{i=1}^{n}Y_{i}>n/2|X^{n}=x^{n}\right) \end{array}$$

(25) $$\begin{array}{cc} \; & =2^{1-n}\cdot \mathrm{Pr}\left(\sum_{i=1}^{n}Y_{i}>n/2|W_{H}\left(X^{n}\right)=W_{H}\left(x^{n}\right)\right) \end{array}$$

(26) $$\begin{array}{cc} \; & =:2^{1-n}\cdot r_{n}\left(W_{H}\left(x^{n}\right)\right), \end{array}$$

where Equation (25) follows from symmetry. Evidently, $r_{n}\left(w\right)$ is an increasing function of w∈{0,1,…,n}. Indeed, let Bin(n,δ) be a binomial r.v. of *n* trials and success probability δ. Then, for any w≤n−1, as δ≤1/2,

$$\begin{array}{cc} \; & r_{n}\left(w+1\right) \end{array}$$

(27) $$\begin{array}{cc} \; & =\mathrm{Pr}\left(\mathrm{Bin}(w+1,1-\delta )+\mathrm{Bin}(n-w-1,\delta )>n/2\right) \end{array}$$

(28) $$\begin{array}{cc} \; & =\mathrm{Pr}\left(\mathrm{Bin}(w,1-\delta )+\mathrm{Bin}(1,1-\delta )+\mathrm{Bin}(n-w-1,\delta )>n/2\right) \end{array}$$

(29) $$\begin{array}{cc} \; & \geq \mathrm{Pr}\left(\mathrm{Bin}(w,1-\delta )+\mathrm{Bin}(1,\delta )+\mathrm{Bin}(n-w-1,\delta )>n/2\right) \end{array}$$

(30) $$\begin{array}{cc} \; & =\mathrm{Pr}\left(\mathrm{Bin}(w,1-\delta )+\mathrm{Bin}(n-w,\delta )>n/2\right) \end{array}$$

(31) $$\begin{array}{cc} \; & =r_{n}\left(w\right), \end{array}$$

where, in each of the above probabilities, the summation is over an independent binomial r.v. Hence, we deduce that, whenever $\mathrm{Maj}(Y^{n})=1$ (resp. $\mathrm{Maj}(Y^{n})=0$), the optimal guessing strategy is by decreasing (resp. increasing) Hamming weight (with arbitrary order for inputs of equal Hamming weight).

We can now turn to evaluate the guessing-moment for the optimal strategy given the majority of $Y^{n}$. Let Mn,w:=∑v=0wnv for w∈{0,1,…,n}. From symmetry,

(32) $$\begin{array}{cc} G_{s}\left(X^{n}|\mathrm{Maj}\left(Y^{n}\right)\right) & =G_{s}\left(X^{n}|\mathrm{Maj}\left(Y^{n}\right)=1\right) \end{array}$$

(33) =∑w=0nnw21−nrn(w)∑i=Mn,w−1+1Mn,wis

where $M_{n,-1}:=0$. Thus,

(34) Gs(Xn∣Maj(Yn))≥∑w=0nnw21−nrn(w)Mn,w−1s

(35) $$\begin{array}{cc} \; & =2^{sn+1}\cdot E\left[r_{n}\left(W\right){\left(\frac{M_{n,W-1}}{2^{n}}\right)}^{s}\right] \end{array}$$

(36) $$\begin{array}{cc} \; & =2^{sn+1}\cdot E\left[r_{n}\left(W\right)\mathrm{Pr}{\left(W^{\prime }\leq W-1\right)}^{s}\right], \end{array}$$

where $W,W^{\prime }∼\mathrm{Bin}\left(n,1/2\right)$ and is independent. For evaluating the asymptotic behavior (for large *n*) of this expression, we note that the Berry–Esseen central-limit theorem ([31], Chapter XVI.5, Theorem 2) leads to (see, e.g., Reference [28], proof of Lemma 15)

(37) $$r_{n}\left(w\right)=Q\left(\beta \cdot \frac{2}{\sqrt{n}}\left[\frac{n}{2}-w\right]\right)+\frac{a_{\delta }}{\sqrt{n}},$$

for some universal constant $a_{\delta }$. Using the Berry–Esseen central-limit theorem again, we have that $\frac{2}{\sqrt{n}}\left(\frac{n}{2}-W^{\prime }\right)\overset{d}{\to }Z$, where Z∼N(0,1) and $\overset{d}{\to }$ denote convergence in distribution. Thus for a given *w*,

(38) $$\begin{array}{cc} \mathrm{Pr}\left(W^{\prime }\leq w-1\right) & =1-\mathrm{Pr}\left(\frac{2}{\sqrt{n}}\left(\frac{n}{2}-W^{\prime }\right)\geq \frac{2}{\sqrt{n}}\left(\frac{n}{2}-w-1\right)\right) \end{array}$$

(39) $$\begin{array}{cc} \; & =1-Q\left(\frac{2}{\sqrt{n}}\left(\frac{n}{2}-w-1\right)\right)-\frac{a_{1/2}}{\sqrt{n}} \end{array}$$

(40) $$\begin{array}{cc} \; & =1-Q\left(\frac{2}{\sqrt{n}}\left(\frac{n}{2}-w\right)\right)-O\left(\frac{1}{\sqrt{n}}\right), \end{array}$$

where the last equality follows from the fact that $|Q^{\prime }\left(t\right)|\leq \frac{1}{\sqrt{2\pi }}$ for all t∈R. Using the Berry–Esseen theorem once again, we have that $\frac{2}{\sqrt{n}}\left(\frac{n}{2}-w\right)\overset{d}{\to }Z$. Hence, Portmanteau’s lemma (e.g., Reference [31], Chapter VIII.1, Theorem 1) and the fact the Q(t) is continuous and bounded result in the following:

(41) $$G_{s}\left(X^{n}|\mathrm{Maj}\left(Y^{n}\right)\right)\geq 2^{sn+1}\cdot E\left[Q\left(\beta N\right)\cdot {\left(1-Q(N)\right)}^{s}\right]+O\left(\frac{1}{n^{s/2}}\right).$$

Similarly to Equation (34), the upper bound

(42) Gs(Xn∣Maj(Yn))≤∑w=0nnw21−nrn(w)Mws,

holds, and a similar analysis leads to an expression which asymptotically coincides with the right-hand side (r.h.s.) of Equation (41). The result then follows from Equation (5) and Lemma 1. □

### 3.3. Proofs of the Lower Bounds on $\gamma_{s,k}\left(\delta \right)$

To prove Theorem 3, we first prove the following maximum entropy result. With a standard abuse of notation, we will write the guessing-moment and the entropy of a random variable as functions of its distribution.

**Lemma** **2.** *The maximal entropy under guessing-moment constraint satisfies*

(43) $$\max_{P:G_{s}\left(P\right)=g}H\left(P\right)=\log\left(e^{1/s}s^{(1-s)/s}\cdot G_{s}^{1/s}\left(P\right)\cdot \Gamma \left(\frac{1}{s}\right)\right)+o\left(1\right),$$

*where o(1) vanishes as g→∞.*

**Proof.**  To solve the maximum entropy problem ([32], Chapter 12) in Equation (43) (note that the support of *P* is only restricted to be countable), we first relax the constraint $G_{s}\left(P\right)=g$ to

(44) $$\sum_{i=1}^{\infty }P\left(i\right)\cdot i^{s}=g,$$

i.e., we omit the requirement that {P(i)} is a decreasing sequence. Assuming momentarily that the entropy is measured in nats, it is easily verified (e.g., using the theory of exponential families ([33], Chapter 3) or by Lagrange duality ([34], Chapter 5)) that the entropy maximizing distribution is

(45) $$P_{\lambda }\left(i\right):=\frac{\exp(-\lambda i^{s})}{Z(\lambda )}$$

for $i\in N_{+}$, where $Z\left(\lambda \right):=\sum_{i=1}^{\infty }\exp\left(-\lambda i^{s}\right)$ is the *partition function* and λ>0 is chosen such that $\sum_{i=1}^{\infty }P_{\lambda }\left(i\right)\cdot i^{s}=g$. Evidently, $P_{\lambda }\left(i\right)$ is in decreasing order (and so is $G_{s}\left(P_{\lambda }\right)=g$) and is therefore the solution to Equation (43). The resulting maximum entropy is then given in a parametric form as

(46) $$H\left(P_{\lambda }\right)=\lambda G_{s}\left(P_{\lambda }\right)+\ln Z\left(\lambda \right).$$

Evidently, if $g=G_{s}\left(P_{\lambda }\right)\to \infty$, then λ→0. In this case, we may approximate the limit of the partition function as λ→0 by a Riemann integral. Specifically, by the monotonicity of $e^{-\lambda i^{s}}$ in i∈N,

(47) $$\begin{array}{cc} Z(\lambda ) & =\sum_{i=1}^{\infty }e^{-\lambda i^{s}} \end{array}$$

(48) $$\begin{array}{cc} \; & =\frac{1}{2}\left(\sum_{i=-\infty }^{\infty }\exp\left(-{\left(\frac{\left|i\right|}{\lambda^{-1/s}}\right)}^{s}\right)-1\right) \end{array}$$

(49) $$\begin{array}{cc} \; & \geq \frac{1}{2}\left(\int_{-\infty }^{\infty }\exp\left(-{\left(\frac{\left|t\right|}{\lambda^{-1/s}}\right)}^{s}\right)dt-1\right) \end{array}$$

(50) $$\begin{array}{cc} \; & =\frac{1}{s}\lambda^{-1/s}\cdot \Gamma \left(\frac{1}{s}\right)-\frac{1}{2}, \end{array}$$

where the last equality follows from the definition of the Gamma function (see Theorem 3) or from the identification of the integral as an unnormalized generalized Gaussian distribution of zero mean, scale parameter $\lambda^{-1/s}$, and shape parameter *s* [35]. Further, by the convexity of $e^{-\lambda t^{s}}$ in $t\in R_{+}$, Jensen’s inequality implies that

(51) $$e^{-\lambda i^{s}}\leq \int_{-i-1/2}^{i+1/2}\exp\left(-{\lambda |t|}^{s}\right)dt$$

for every i≥1 (the r.h.s. can be considered as averaging over a uniform random variable [i−1/2,i+1/2]) and so, similarly to Equation (50),

(52) $$\begin{array}{cc} Z(\lambda ) & \leq \frac{1}{2}\left(\int_{-\infty }^{\infty }\exp\left(-{\left(\frac{\left|t\right|}{\lambda^{-1/s}}\right)}^{s}\right)dt\right). \end{array}$$

Therefore,

(53) $$Z\left(\lambda \right)=\left(1+a_{\lambda }\right)\cdot \frac{1}{s}\lambda^{-1/s}\cdot \Gamma \left(\frac{1}{s}\right)$$

where $a_{\lambda }\to 0$ as λ→0. In the same spirit,

(54) $$\begin{array}{cc} G_{s}\left(P_{\lambda }\right) & =\sum_{i=1}^{\infty }i^{s}\cdot \frac{\exp(-\lambda i^{s})}{Z(\lambda )} \end{array}$$

(55) $$\begin{array}{cc} \; & =\frac{\int_{0}^{\infty }t^{s}\exp\left(-{\left(\frac{\left|t\right|}{\lambda^{-1/s}}\right)}^{s}\right)dt+b_{\lambda }}{\left(1+a_{\lambda }\right)\frac{1}{s}\lambda^{-1/s}\cdot \Gamma \left(\frac{1}{s}\right)} \end{array}$$

(56) $$\begin{array}{cc} \; & =\frac{\frac{1}{s}\lambda^{-\frac{s+1}{s}}\cdot \Gamma \left(\frac{s+1}{s}\right)+b_{\lambda }}{\left(1+a_{\lambda }\right)\frac{1}{s}\lambda^{-1/s}\cdot \Gamma \left(\frac{1}{s}\right)} \end{array}$$

(57) $$\begin{array}{cc} \; & =\frac{\frac{1}{s^{2}}\lambda^{-\frac{s+1}{s}}\cdot \Gamma \left(\frac{1}{s}\right)+b_{\lambda }}{\left(1+a_{\lambda }\right)\frac{1}{s}\lambda^{-1/s}\cdot \Gamma \left(\frac{1}{s}\right)} \end{array}$$

(58) $$\begin{array}{cc} \; & =\frac{1}{s\lambda }\left(1+c_{\lambda }\right), \end{array}$$

where in Equation (56), $b_{\lambda }\to 0$ as λ→0; in Equation (57), the identity Γ(t+1)=tΓ(t) for $t\in R_{+}$ was used; and in Equation (58), $c_{\lambda }\to 0$ as λ→0.

Returning to measure entropy in bits, we thus obtain that, for any distribution *P*,

(59) $$H\left(P\right)\leq \log\left(\frac{e^{1/s}}{s}s^{1/s}\cdot G_{s}^{1/s}\left(P\right)\cdot \Gamma \left(\frac{1}{s}\right)\right)+o\left(1\right),$$

or, equivalently,

(60) $$G_{s}\left(P\right)\geq \Psi_{s}\cdot 2^{sH(P)}\cdot \left(1+o\left(1\right)\right),$$

where $\Psi_{s}:=e^{-1}\cdot \frac{s^{s-1}}{\Gamma^{s}\left(\frac{1}{s}\right)}$ and o(1) is a vanishing term as $G_{s}\left(P\right)\to \infty$. In the same spirit, Equation (60) holds whenever H(P)→∞. □

**Remark** **2.** *In Reference [8], the maximum-entropy problem was studied for s=1. In this case, the maximum-entropy distribution is readily identified as the geometric distribution. The proof above generalizes that result to any s>0.*

**Proof** **of** **Theorem** **3.** Assume that *f* is taken from a sequence of functions which achieves the minimum in Equation (5). Using Lemma 2 when conditioning on $f\left(Y^{n}\right)=b^{k}$ for each of possible $b^{k}$, we get (see a rigorous justification to Equation (61) in Appendix A)

(61) $$\begin{array}{cc} G_{s}\left(X^{n}|f\left(Y^{n}\right)\right) & \geq \ell_{n}\cdot \Psi_{s}\cdot \sum_{b^{k}\in {\left\{0,1\right\}}^{k}}\mathrm{Pr}\left(f\left(Y^{n}\right)=b^{k}\right)\cdot 2^{sH(X^{n}|f\left(Y^{n}\right)=b^{k})} \end{array}$$

(62) $$\begin{array}{cc} \; & \geq \ell_{n}\cdot \Psi_{s}\cdot 2^{sH(X^{n}|f\left(Y^{n}\right))} \end{array}$$

(63) $$\begin{array}{cc} \; & \geq \ell_{n}\cdot \Psi_{s}\cdot 2^{s[n-k{\left(1-2\delta \right)}^{2}]} \end{array}$$

where in Equation (61), $\ell_{n}≐1$ and Equation (62) follows from Jensen’s inequality. For k=1, the bound in Equation (63) is directly related to the Boolean function conjecture [22] and may be proved in several ways, e.g., using Mrs. Gerber’s Lemma ([36], Theorem 1); see ([23], Section IV), References [27,37]. For general k≥1, the bound $H\left(X^{n}|f\left(Y^{n}\right)\right)\geq n-k{\left(1-2\delta \right)}^{2}$ was established in Reference ([27], Corollary 1). □

Before presenting the proof of the Fourier-based bound, we briefly remind the reader of the basic definitions and results of Fourier analysis of Boolean functions [21], and to that end, it is convenient to replace the binary alphabet {0,1} by {−1,1}. An inner product between two real-valued functions on the Boolean cube $f,g:{\left\{-1,1\right\}}^{n}\to R$ is defined as

(64) $$\langle f,g\rangle :=E\left(f\left(X^{n}\right)g\left(X^{n}\right)\right),$$

where $X^{n}\in {\left\{-1,1\right\}}^{n}$ is a uniform Bernoulli vector. A *character* associated with a set of coordinates S⊆[n]:={1,2,…,n} is the Boolean function $x^{S}:=\prod_{i\in S}x_{i}$, where by convention, $x^{\emptyset }:=1$. It can be shown ([21], Chapter 1) that the set of all characters forms an orthonormal basis with respect to the inner product (Equation (64)). Furthermore,

(65) $$f\left(x^{n}\right)=\sum_{S\subseteq [n]}{\hat{f}}_{S}\cdot x^{S},$$

where ${\left\{{\hat{f}}_{S}\right\}}_{S\subseteq [n]}$ are the *Fourier coefficients* of *f*, given by ${\hat{f}}_{S}=\langle x^{S},f\rangle =E\left(X^{S}\cdot f\left(X^{n}\right)\right)$. *Plancherel’s identity* then states that $\langle f,g\rangle =E\left(f\left(X^{n}\right)g\left(X^{n}\right)\right)=\sum_{S\in [n]}{\hat{f}}_{S}{\hat{g}}_{S}$. The *p* norm of a function *f* is defined as ${∥f∥}_{p}:=[E|f\left(X^{n}\right){{|}^{p}]}^{1/p}$.

The *noise operator* operating on a Boolean function *f* is defined as

(66) $$T_{\rho }f\left(x^{n}\right)=E\left(f\left(Y^{n}\right)|X^{n}=x^{n}\right)$$

where ρ:=1−2δ is the *correlation parameter*. The noise operator has a smoothing effect on the function which is captured by the so-called hypercontractivity theorems. Specifically, we shall use the following version.

**Theorem** **5** ([21], p. 248)**.** *Let $f:{\left\{-1,1\right\}}^{n}\to R$ and 0≤ρ≤1. Then, $∥T_{\rho }{f∥}_{2}\leq {∥f∥}_{\rho^{2}+1}$.*

With the above, we can prove Theorem 4.

**Proof** **of** **Theorem** **4.** From Bayes law (recall that $f\left(x^{n}\right)\in \left\{-1,1\right\}$)

(67) $$\mathrm{Pr}\left(X^{n}=x^{n}|f\left(Y^{n}\right)=b\right)=2^{-(n+1)}\cdot \frac{1+bT_{\rho }f\left(x^{n}\right)}{\mathrm{Pr}(f\left(Y^{n}\right)=b)},$$

and from the law of total expectation

(68) $$G_{s}\left(X^{n}|f\left(Y^{n}\right)\right)=\mathrm{Pr}\left(f\left(Y^{n}\right)=1\right)\cdot G_{s}\left(X^{n}|f\left(Y^{n}\right)=1\right)+\mathrm{Pr}\left(f\left(Y^{n}\right)=-1\right)\cdot G_{s}\left(X^{n}|f\left(Y^{n}\right)=-1\right).$$

Let us denote ${\hat{f}}_{\phi }=Ef\left(X^{n}\right)$ and $g:=f-{\hat{f}}_{\phi }$ and abbreviate ${\mathrm{ORD}}_{f}\left(x^{n}\right):={\mathrm{ORD}}_{X^{n}|f\left(Y^{n}\right)}\left(x^{n}|1\right)$. Then, the first addend on the r.h.s. of Equation (68) is given by

(69) $$\begin{array}{cc} \mathrm{Pr}\left(f\left(Y^{n}\right)=1\right)\cdot G_{s}\left(X^{n}|f\left(Y^{n}\right)=1\right) & =2^{-(n+1)}\sum_{x^{n}}\left(1+{\hat{f}}_{\phi }+T_{\rho }g\left(x^{n}\right)\right)\cdot {\mathrm{ORD}}_{T_{\rho }g}^{s}\left(x^{n}\right) \end{array}$$

(70) $$\begin{array}{cc} \; & =\frac{\left(1+{\hat{f}}_{\phi }\right)}{2}\cdot E\left({\mathrm{ORD}}_{T_{\rho }g}^{s}\left(X^{n}\right)\right)+\frac{1}{2}\langle T_{\rho }g,{\mathrm{ORD}}_{T_{\rho }g}^{s}\rangle \end{array}$$

(71) $$\begin{array}{cc} \; & =\frac{\left(1+{\hat{f}}_{\phi }\right)}{2}\cdot K_{s}\left(2^{n}\right)+\frac{1}{2}\langle T_{\rho }g,{\mathrm{ORD}}_{T_{\rho }g}^{s}\rangle \end{array}$$

(72) $$\begin{array}{cc} \; & =\frac{\left(1+{\hat{f}}_{\phi }\right)}{2}\cdot \ell_{n}\cdot \frac{2^{sn}}{s+1}+\frac{1}{2}\langle T_{\rho }g,{\mathrm{ORD}}_{T_{\rho }g}^{s}\rangle , \end{array}$$

where, in the last equality, $\ell_{n}≐1$ (Lemma 1). Let λ∈[0,1], and denote $\rho_{1}:=\rho^{\lambda }$ and $\rho_{2}=\rho^{1-\lambda }$. Then, the inner-product term in Equation (72) is upper bounded as

(73) $$\begin{array}{cc} \left|\langle T_{\rho }g,{\mathrm{ORD}}_{T_{\rho }g}^{s}\rangle \right| & =\left|\langle T_{\rho_{1}}g,T_{\rho_{2}}{\mathrm{ORD}}_{T_{\rho }g}^{s}\rangle \right| \end{array}$$

(74) $$\begin{array}{cc} \; & \leq ∥T_{\rho_{1}}{g∥}_{2}\cdot {∥T_{\rho_{2}}{\mathrm{ORD}}_{T_{\rho }g}^{s}∥}_{2} \end{array}$$

(75) $$\begin{array}{cc} \; & \leq \rho_{1}\cdot \sqrt{1-{\hat{f}}_{\phi }^{2}}\cdot {∥T_{\rho_{2}}{\mathrm{ORD}}_{T_{\rho }g}^{s}∥}_{2} \end{array}$$

(76) $$\begin{array}{cc} \; & \leq \rho_{1}\cdot \sqrt{1-{\hat{f}}_{\phi }^{2}}\cdot {∥{\mathrm{ORD}}_{T_{\rho }g}^{s}∥}_{1+\rho_{2}^{2}} \end{array}$$

(77) $$\begin{array}{cc} \; & =\rho_{1}\cdot \sqrt{1-{\hat{f}}_{\phi }^{2}}\cdot {\left(K_{(1+\rho_{2}^{2})s}\left(2^{n}\right)\right)}^{1/(1+\rho_{2}^{2})} \end{array}$$

(78) $$\begin{array}{cc} \; & =\rho_{1}\cdot \sqrt{1-{\hat{f}}_{\phi }^{2}}\cdot {\left[k_{n}\cdot \frac{1}{(1+\rho_{2}^{2})s+1}\right]}^{1/(1+\rho_{2}^{2})}\cdot 2^{sn}, \end{array}$$

where Equation (73) holds since $T_{\rho }$ is a self-adjoint operator and Equation (74) follows from the Cauchy–Schwarz inequality. To justify Equation (75), we note that

(79) $$\begin{array}{cc} ∥T_{\rho }{g∥}_{2}^{2} & =\langle T_{\rho }g,T_{\rho }g\rangle \end{array}$$

(80) $$\begin{array}{cc} \; & =\sum_{S\in [n]}\rho^{2|S|}{\hat{g}}_{S}^{2} \end{array}$$

(81) $$\begin{array}{cc} \; & =\sum_{S\in [n]\backslash \phi }\rho^{2|S|}{\hat{f}}_{S}^{2} \end{array}$$

(82) $$\begin{array}{cc} \; & \leq \rho^{2}\cdot \left(1-{\hat{f}}_{\phi }^{2}\right), \end{array}$$

where Equation (80) follows from Plancherel’s identity, Equation (81) is since ${\hat{g}}_{S}={\hat{f}}_{S}$ for all S≠ϕ and ${\hat{g}}_{\phi }=0$, and Equation (82) follows from $\sum_{S\in [n]}{\hat{f}}_{S}^{2}={∥f∥}_{2}^{2}=Ef^{2}=1$. Equation (76) follows from Theorem 5, and in Equation (78), $k_{n}≐1$. The second addend on the r.h.s. of Equation (68) can be bounded in the same manner. Hence,

(83) $$\begin{array}{cc} G_{s}\left(X^{n}|f\left(Y^{n}\right)\right) & \geq \max_{0\leq \lambda \leq 1}2^{sn}\cdot \left[\ell_{n}\cdot \frac{1}{s+1}-\rho^{\lambda }\cdot \sqrt{1-{\hat{f}}_{\phi }^{2}}\cdot {\left[k_{n}\cdot \frac{1}{(1+\rho^{2(1-\lambda )})s+1}\right]}^{1/(1+\rho^{2(1-\lambda )})}\right] \end{array}$$

(84) $$\begin{array}{cc} \; & \geq \max_{0\leq \lambda \leq 1}2^{sn}\cdot \left[\ell_{n}\cdot \frac{1}{s+1}-\rho^{\lambda }{\left[k_{n}\cdot \frac{1}{(1+\rho^{2(1-\lambda )})s+1}\right]}^{1/(1+\rho^{2(1-\lambda )})}\right] \end{array}$$

(85) $$\begin{array}{cc} \; & \to 2^{sn}\cdot \max_{0\leq \lambda \leq 1}\left[\frac{1}{s+1}-\frac{\rho^{\lambda }}{{\left[(1+\rho^{2(1-\lambda )})s+1\right]}^{1/(1+\rho^{2(1-\lambda )})}}\right] \end{array}$$

as n→∞. □

We close this section with the following proof of Proposition 2:

**Proof** **of** **Proposition** **2.** Let $I=(i_{1},\ldots ,i_{L})$ be a vector of indices in [n] such that $1\leq i_{1}<i_{2}<\cdots <i_{L}\leq n$, and let $x^{n}\left(I\right)=\left(x_{i_{1}},\ldots ,x_{i_{L}}\right)$ be the components of $x^{n}$ in those indices. Further, let ${\left\{f^{\left(n\right)}\right\}}_{n=1}^{\infty }\in F_{k}$. Then, it holds that

(86) $$\mathrm{Pr}\left[X^{n}=x^{n},f^{\left(n\right)}\left(Y^{n}\right)\right]=\prod_{j=1}^{k}\mathrm{Pr}\left[X^{n}\left(I_{j}\right)=x^{n}\left(I_{j}\right),f_{j}^{\left(n\right)}\left(y^{n}\right)\right],$$

as well as

(87) $${\mathrm{ORD}}_{X^{n}|f^{\left(n\right)}\left(Y^{n}\right)}\left(x^{n}|b^{k}\right)\geq \prod_{j=1}^{k}\left[{\mathrm{ORD}}_{X^{n}\left(I_{j}\right)|f_{j}^{\left(n\right)}\left(Y^{n}\right)}\left(x^{n}\left(I_{j}\right)|b_{j}\right)-1\right].$$

Hence,

(88) $$\begin{array}{cc} G_{s}\left(X^{n}|f^{\left(n\right)}\left(Y^{n}\right)\right) & \geq \prod_{j=1}^{k}\left[G_{s}^{\left(n\right)}\left(X^{n}\left(I_{j}\right)|f_{j}^{\left(n\right)}\left(Y^{n}\right)\right)-1\right] \end{array}$$

and the stated bound is deduced after taking limits and normalizing by $G_{s}\left(X^{n}\right)≐\frac{2^{sn}}{s+1}$. □

## 4. Guessing Ratio for a General Binary Input Channel

In this section, we consider the guessing ratio for general channels with a uniform binary input. The lower bound of Theorem 3 can be easily generalized to this case. To that end, consider the SDPI constant [38,39] of the reverse channel $\left(P_{Y},P_{X|Y}\right)$, given by

(89) $$\eta \left(P_{Y},P_{Y|X}\right):=\underset{Q_{Y}:Q_{Y}\neq P_{Y}}{\sup}\frac{D(Q_{X}||P_{X})}{D(Q_{Y}||P_{Y})},$$

where $Q_{X}$ is the *X*-marginal of $Q_{Y}\circ P_{X|Y}$. As was shown in Reference ([40], Theorem 2), the SDPI constant of $\left(P_{Y},P_{X|Y}\right)$ is also given by

(90) $$\eta \left(P_{Y},P_{Y|X}\right)=\underset{P_{W|Y}:W-Y-X,\;I\left(W;Y\right)>0}{\sup}\frac{I(W;X)}{I(W;Y)}.$$

**Theorem** **6.** *We have*

(91) $$\gamma_{s,k}\left(P_{X},P_{Y|X}\right)\geq e^{-1}\cdot \frac{s^{s-1}\cdot \left(s+1\right)}{\Gamma^{s}\left(\frac{1}{s}\right)}\cdot 2^{-s\cdot k\cdot \eta (P_{Y},P_{X|Y})}.$$

**Proof.**  See Appendix A. □

**Remark** **3.** *The bound for the BSC case (Theorem 3) is indeed a special case of Theorem 6 as the reverse BSC channel is also a BSC with uniform input and the same crossover probability. For BSCs, it is well known that the SDPI constant is ${\left(1-2\delta \right)}^{2}$ ([38], Theorem 9).*

Next, we consider in more detail the case where the observation channel is a BEC. We restrict the discussion to the case of a single bit of help, k=1.

### 4.1. Binary Erasure Channel

Suppose that $Y^{n}\in {\left\{0,1,e\right\}}^{n}$ is obtained from $X^{n}$ by erasing each bit independently with probability ϵ∈[0,1]. As before, Bob observes the channel output $Y^{n}$ and can send one bit $f:{\left\{0,1,e\right\}}^{n}\to \left\{0,1\right\}$ to Alice, who wishes to guess $X^{n}$. With a slight abuse of notation, the guessing ratio in Equation (5) will be denoted by $\gamma_{s}\left(\epsilon \right)$.

**Figure 4:**
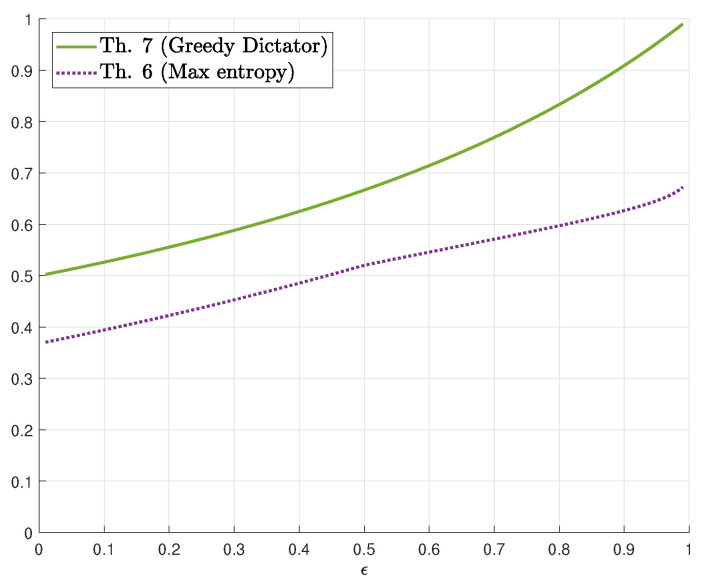
Bounds on $\gamma_{s}\left(\delta \right)$ for s=1 as a function of ϵ∈[0,1].

To compute the lower bound of Theorem 6, we need to find the SDPI constant associated with the reverse channel, which is easily verified to be

(92) $$P_{X|Y=y}\left(x\right)=\left\{\begin{array}{cc} 𝟙(x=y), & y=0\;\mathrm{or}\;y=1\\ \mathrm{Ber}(1/2), & y=e, \end{array}\right.$$

with an input distribution $P_{Y}=\left(\frac{1-\epsilon }{2},\epsilon ,\frac{1-\epsilon }{2}\right)$. Letting $Q_{Y}\left(y\right)=q_{y}$ for y∈{0,1,e} yields $Q_{X}\left(x\right)=q_{x}+\frac{q_{e}}{2}$ for x∈{0,1}. The computation of $\eta (P_{Y},P_{X|Y})$ is now a simple three-dimensional constrained optimization problem. We plotted the resulting lower bound for s=1 in Figure 4.

Let us now turn to upper bounds and focus for simplicity on the average guessing time, i.e., the guessing-moment for s=1. To begin, let *S* represent the set of indices of the symbols that were not erased, i.e., i∈S if and only if $Y_{i}\neq e$. Any function $f:{\left\{0,1,e\right\}}^{n}\to \left\{0,1\right\}$ is then uniquely associated with a set of Boolean functions ${\left\{f_{S}\right\}}_{S\in [n]}$, where $f_{S}:{\left\{0,1\right\}}^{\left|S\right|}\to \left\{0,1\right\}$ designates the operation of the function when *S* is the set of non-erased symbols. We also let $\mathrm{Pr}\left(S\right)={\left(1-\epsilon \right)}^{\left|S\right|}\cdot \epsilon^{|S^{c}|}$ be the probability that the non-erased symbols have index set *S*. Then, the joint probability distribution is given by

(93) $$\begin{array}{cc} \mathrm{Pr}(X^{n}=x^{n},f\left(Y^{n}\right)=1) & =\mathrm{Pr}\left(X^{n}=x^{n}\right)\cdot \mathrm{Pr}\left(f\left(Y^{n}\right)=1|X^{n}=x^{n}\right) \end{array}$$

(94) $$\begin{array}{cc} \; & =2^{-n}\cdot \sum_{S\subseteq [n]}\mathrm{Pr}\left(S\right)\cdot \mathrm{Pr}\left(f\left(Y^{n}\right)=1|X^{n}=x^{n},S\right) \end{array}$$

(95) $$\begin{array}{cc} \; & =2^{-n}\cdot \sum_{S\subseteq [n]}\mathrm{Pr}\left(S\right)\cdot f_{S}\left(x^{n}\right), \end{array}$$

and, similarly,

(96) $$\begin{array}{cc} \mathrm{Pr}(X^{n}=x^{n},f\left(Y^{n}\right)=0) & =2^{-n}\cdot \sum_{S\subseteq [n]}\mathrm{Pr}\left(S\right)\cdot \left(1-f_{S}\left(x^{n}\right)\right) \end{array}$$

(97) $$\begin{array}{cc} \; & =2^{-n}-2^{-n}\cdot \sum_{S\subseteq [n]}\mathrm{Pr}\left(S\right)\cdot f_{S}\left(x^{n}\right). \end{array}$$

In accordance with Proposition 1, the optimal guessing order given that $f(Y^{n})=0$ is reversed to the optimal guessing order when $f(Y^{n})=1$. It is also apparent that the posterior probability is determined by a mixture of $2^{n}$ different Boolean functions ${\left\{f_{S}\right\}}_{S\in [n]}$. This may be contrasted with the BSC case, in which the posterior is determined by a *single* Boolean function (though with noisy input).

A seemingly natural choice is a *greedy dictator* function, for which $f(Y^{n})$ sends the first non-erased bit. Concretely, letting

(98) $$k\left(y^{n}\right):=\left\{\begin{array}{cc} n+1, & y^{n}=e^{n}\\ \min\left\{i:y_{i}\neq e\right\}, & \mathrm{otherwise} \end{array}\right.,$$

the *greedy dictator* function is defined by

(99) $$\text{G-Dict}\left(y^{n}\right):=\left\{\begin{array}{cc} \mathrm{Ber}(1/2), & y^{n}=e^{n}\\ y_{k(y^{n})}, & \mathrm{otherwise} \end{array}\right.,$$

where Ber(α) is a Bernoulli r.v. of success probability α. From an analysis of the posterior probability, it is evident that, conditioned on $f(Y^{n})=0$, an optimal guessing order must satisfy that $x^{n}$ is guessed before $z^{n}$ whenever

(100) $$\sum_{i=1}^{n}\epsilon^{i-1}\cdot x_{i}\leq \sum_{i=1}^{n}\epsilon^{i-1}\cdot z_{i},$$

(see Appendix A for a proof of Equation (100)). This rule can be loosely thought of as comparing the “base 1/ϵ expansion” of $x^{n}$ and $z^{n}$. Furthermore, when ϵ is close to 1, then the optimal guessing order tends toward a *minimum Hamming weight* rule (or maximum Hamming weight in case f=1).

The greedy dictator function is “locally optimal” when ϵ∈[0,1/2], in the following sense:

**Proposition** **3.** *If ϵ∈[0,1/2], then an optimal guessing order conditioning on $\text{G-Dict}(Y^{n})=0$ (resp. $\text{G-Dict}(Y^{n})=1$) is lexicographic (reverse lexicographic). Also, given lexicographic (resp. reverse lexicographic) order when the received bit is 0 (resp. 1), the optimal function f is a greedy dictator.*

**Proof.**  See Appendix A. □

The guessing ratio of the greedy dictator function can be evaluated for s=1, and the analysis leads to the following upper bound:

**Theorem** **7.** *For s=1, the guessing ratio is upper bounded as*

(101) $$\gamma_{1}\left(\epsilon \right)\leq \frac{1}{2-\epsilon },$$

*and the r.h.s. is achieved with equality by the greedy dictator function in Equation (99) for ϵ∈[0,1/2].*

**Proof.**  See Appendix A. □

The upper bound of Theorem 7 is plotted in Figure 4. Based on Proposition 3 and numerical computations for moderate values of *n*, we conjecture:

**Conjecture.** **1.** *Greedy dictator functions attain $\gamma_{s}\left(\epsilon \right)$ for the BEC.*

Supporting evidence for this conjecture include the local optimality property stated in Proposition 3 (although there are other locally optimal choices) as well as the following heuristic argument: Intuitively, Bob should reveal as much as possible regarding the bits he has seen and as little as possible regarding the erasure pattern. So, it seems reasonable to find a smallest possible set of balanced functions from which to choose all the functions $f_{S}$, so that they coincide as much as possible. Greedy dictator is a greedy solution to this problem: it uses the function $x_{1}$ for half of the erasure patterns, which is the maximum possible. Then, it uses the function $x_{2}$ for half of the remaining patterns, and so on. Indeed, we were not able to find a better function than G-Dict for small values of *n*.

However, applying standard techniques in attempt to prove Conjecture 1 has not been fruitful. One possible technique is induction. For example, assume that the optimal functions for dimension n−1 are $f_{S}^{\left(n-1\right)}$. Then, it might be perceived that there exists a bit, say $x_{1}$, such that the optimal functions for dimension *n* satisfy $f_{S}^{\left(n\right)}=f_{S}^{\left(n-1\right)}$ if $x_{1}$ is erased; in that case, it remains only to determine $f_{S}^{\left(n\right)}$ when $x_{1}$ is not erased. However, observing Equation (95), it is apparent that the optimal choice of $f_{S}^{\left(n\right)}$ should satisfy two contradicting goals—on the one hand, to match the order induced by

(102) $$\sum_{S\subseteq [n]:1\notin S}\mathrm{Pr}\left(S\right)\cdot f_{S}\left(x^{n}\right)$$

and, on the other hand, to minimize the average guessing time of

(103) $$\sum_{S\subseteq [n]:1\in S}\mathrm{Pr}\left(S\right)\cdot f_{S}\left(x^{n}\right).$$

It is easy to see that taking a greedy approach toward satisfying the second goal would result in $f_{S}^{\left(n\right)}\left(x^{n}\right)=x_{1}$ if 1∈S and performing the recursion steps would indeed lead to a greedy dictator function. Interestingly, taking a greedy approach toward satisfying the first goal would also lead to a greedy dictator function, but one which operates on a cyclic permutation of the inputs (specifically, Equation (99) applied to $\left(y_{2}^{n},y_{1}\right)$). Nonetheless, it is not clear that choosing ${\left\{f_{S}^{\left(n\right)}\right\}}_{S:1\in S}$ with some loss in the average guessing time induced by Equation (103) could not lead to a gain in the second goal (matching the order of Equation (102)), which outweighs that loss.

Another possible technique is majorization. It is known that, if one probability distribution majorizes another, then all the nonnegative guessing-moments of the first are no greater than the corresponding moments of the second ([29], Proposition 1). (The proof in Reference [29] is only for s=1, but it is easily extended to the general s>0 case.) Hence, one approach toward identifying the optimal function could be to try and find a function in which induced posterior distributions majorize the corresponding posteriors that induces by any other functions with the same bias (it is of course not clear that such a function even exists). This approach unfortunately fails for the greedy dictator. For example, the posterior distributions induced by setting $f_{S}$ to be majority functions are not always majorized by those induces by the greedy dictator (although they seem to be “almost” majorized) even though the average guessing time of greedy dictator is lower (this happens, e.g., for n=5 and ϵ=0.4). In fact, the guessing moments for greedy dictator seem to be better than these of majority irrespective of the value of *s*.

## Abbreviations

The following abbreviations are used in this manuscript:

BEC	binary erasure channel
BSC	binary symmetric channel
i.i.d.	independent and identically distributed
r.h.s.	right-hand side
r.v.	random variable
SDPI	strong data-processing inequality
w.l.o.g.	without loss of generality

## Appendix A. Miscellaneous Proofs

**Proof** **of** **Proposition** **1.** The claim that random functions do not improve beyond deterministic ones follows directly from that property that conditioning reduces guessing-moment ([1], Corollary 1). Monotonicity follows from the fact that Bob can always simulate a noisier channel. Now, if δ=1/2, then $X^{n}$ and $Y^{n}$ are independent and $G_{s}\left(X^{n}|f\left(Y^{n}\right)\right)=G_{s}\left(X^{n}\right)≐\frac{2^{sn}}{s+1}$ for any *f* (Lemma 1). For δ=0, let

(A1) $$\gamma_{s,k}^{\left(n\right)}\left(\delta \right):=\min_{f:{\left\{0,1\right\}}^{n}\to {\left\{0,1\right\}}^{k}}\frac{G_{s}\left(X^{n}|f\left(Y^{n}\right)\right)}{G_{s}\left(X^{n}\right)},$$

and let ${\left\{f_{n,k}^{*}\right\}}_{n=1}^{\infty }$ be a sequence of functions such that $f_{n,k}^{*}$ achieves $\gamma_{s,k}^{\left(n\right)}\left(\delta \right)$. We show that $f_{n,k}^{*}$ must satisfy $\mathrm{Pr}\left[f\left(Y^{n}\right)=b^{k}\right]=2^{-k}$ for all $b^{k}\in {\left\{0,1\right\}}^{k}$. If we denote $B^{k}=f_{n,k}^{*}\left(Y^{n}\right)$, then this is equivalent to showing that $\mathrm{Pr}[B_{l}=1|B_{1}=b_{1},\ldots ,B_{l-1}=b_{l-1},B_{l}=b_{l},\ldots B_{k}=b_{k}]=1/2$ for all l∈[k] and $\left(b_{1},\ldots ,b_{l-1},b_{l},\ldots ,b_{k}\right)\in {\left\{0,1\right\}}^{k-1}$. Assume towards contradiction that the optimal function does not satisfy this property for, say, l=k. Let us denote $\mathrm{Pr}\left(f_{n,k}^{*}\left(Y^{n}\right)=b^{k}\right):=q\left(b^{k}\right)$ and assume w.l.o.g. that $q\left(b^{k-1},0\right)>q\left(b^{k-1},1\right)$ for all $b^{k-1}\in {\left\{0,1\right\}}^{k-1}$ (for notational simplicity). Further, let $\bar{q}\left(b^{k-1}\right):=\frac{1}{2}\left[q\left(b^{k-1},0\right)+q\left(b^{k-1},1\right)\right]$. Then,

$$\begin{array}{l} G_{s}\left(X^{n}|f_{n,k}^{*}\left(Y^{n}\right)\right)\\ =\sum_{b^{k-1}\in {\left\{0,1\right\}}^{k-1}}q\left(b^{k-1},0\right)\cdot G_{s}\left(X^{n}|f_{n,k}^{*}\left(Y^{n}\right)=\left(b^{k-1},0\right)\right)+q\left(b^{k-1},1\right)\cdot G_{s}\left(X^{n}|f_{n,k}^{*}\left(Y^{n}\right)=\left(b^{k-1},1\right)\right)\\ =\sum_{b^{k-1}\in {\left\{0,1\right\}}^{k-1}}q\left(b^{k-1},0\right)\cdot K_{s}\left(q\left(b^{k-1},0\right)\cdot 2^{n}\right)+q\left(b^{k-1},1\right)\cdot K_{s}\left(q\left(b^{k-1},1\right)\cdot 2^{n}\right)\\ =2^{-n}\sum_{b^{k-1}\in {\left\{0,1\right\}}^{k-1}}\left(\sum_{i=1}^{q\left(b^{k-1},0\right)\cdot 2^{n}}i^{s}+\sum_{i=1}^{q\left(b^{k-1},1\right)\cdot 2^{n}}i^{s}\right)\\ =2^{-n}\sum_{b^{k-1}\in {\left\{0,1\right\}}^{k-1}}\left(\sum_{i=1}^{\bar{q}\left(b^{k-1}\right)\cdot 2^{n}}i^{s}+\sum_{i=\bar{q}\left(b^{k-1}\right)+1}^{q\left(b^{k-1},0\right)\cdot 2^{n}}i^{s}+\sum_{i=1}^{\bar{q}\left(b^{k-1}\right)\cdot 2^{n}}i^{s}-\sum_{i=q(b^{k-1},1)}^{\bar{q}\left(b^{k-1}\right)\cdot 2^{n}}i^{s}\right)\\ >2^{-(n-1)}\sum_{b^{k-1}\in {\left\{0,1\right\}}^{k-1}}\sum_{i=1}^{\bar{q}\left(b^{k-1}\right)\cdot 2^{n}}i^{s}. \end{array}$$

As equality can be achieved if we modify $f_{n,k}^{*}$ to satisfy $q\left(b^{k-1},0\right)=q\left(b^{k-1},1\right)$ for all $b^{k-1}\in {\left\{0,1\right\}}^{k-1}$, this contradicts the assumed optimality of $f_{n,k}^{*}$. The minimal $G_{s}\left(X^{n}|f\left(Y^{n}\right)\right)$ is thus obtained by any function for which $f\left(Y^{n}\right)\in {\left\{0,1\right\}}^{k}$ is a uniform Bernoulli vector and equals to $K_{s}\left(2^{n-k}\right)≐\frac{2^{s(n-k)}}{s+1}$ (Lemma 1). To prove that the limit in Equation (5) exists, we note that

(A2) $$\begin{array}{cc} G_{s}\left(X^{n+1}\right) & =2^{-(n+1)}\sum_{i=1}^{2^{n+1}}i^{s} \end{array}$$

(A3) $$\begin{array}{cc} \; & =2^{-(n+1)}\sum_{i=1}^{2^{n}}{\left(2i-1\right)}^{s}+{\left(2i\right)}^{s} \end{array}$$

(A4) $$\begin{array}{cc} \; & \geq 2^{s}\cdot 2^{-n}\sum_{i=1}^{2^{n}}{\left(i-1\right)}^{s} \end{array}$$

(A5) $$\begin{array}{cc} \; & =\ell_{n}\cdot 2^{s}\cdot 2^{-n}\sum_{i=1}^{2^{n}}i^{s} \end{array}$$

(A6) $$\begin{array}{cc} \; & =\ell_{n}\cdot 2^{s}\cdot G_{s}\left(X^{n}\right), \end{array}$$

where

(A7) $$\ell_{n}:=\frac{\sum_{i=1}^{2^{n}}{\left(i-1\right)}^{s}}{\sum_{i=1}^{2^{n}}i^{s}}.$$

As before, let ${\left\{f_{n,k}^{*}\right\}}_{n=1}^{\infty }$ be a sequence of functions such that $f_{n,k}^{*}$ achieves $\gamma_{s,k}^{\left(n\right)}\left(\delta \right)$. Denote the order induced by the posterior $\mathrm{Pr}(X^{n}=x^{n}|f_{n,k}^{*}\left(Y^{n}\right)=b^{k})$ as ${\mathrm{ORD}}_{b^{k},n,n}$, $b^{k}\in {\left\{0,1\right\}}^{k}$ and the order induced by $\mathrm{Pr}(X^{n+1}=x^{n+1}|f_{n}^{*}\left(Y^{n}\right)=b^{k})$ as ${\mathrm{ORD}}_{b^{k},n,n+1}$. As before (when breaking ties arbitrarily)

(A8) $${\mathrm{ORD}}_{b^{k},n,n+1}\left(x^{n},0\right)=2{\mathrm{ORD}}_{b^{k},n,n}\left(x^{n}\right)$$

and

(A9) $${\mathrm{ORD}}_{b^{k},n,n+1}\left(x^{n},1\right)=2{\mathrm{ORD}}_{b^{k},n,n}\left(x^{n}\right)-1\leq 2{\mathrm{ORD}}_{b^{k},n,n}\left(x^{n}\right).$$

Thus,

$$\begin{array}{cc} \; & G_{s}\left(X^{n+1}|f_{n+1,k}^{*}\left(Y^{n+1}\right)\right) \end{array}$$

(A10) $$\begin{array}{cc} \; & \leq G_{s}\left(X^{n+1}|f_{n,k}^{*}\left(Y^{n}\right)\right) \end{array}$$

(A11) $$\begin{array}{cc} \; & =\sum_{b^{k}\in {\left\{0,1\right\}}^{k}}\mathrm{Pr}\left(f_{n,k}^{*}\left(Y^{n+1}\right)=b^{k}\right)\cdot G_{s}\left(X^{n+1}|f_{n,k}^{*}\left(Y^{n}\right)=b^{k}\right)\\ \; & \leq \sum_{b^{k}\in {\left\{0,1\right\}}^{k}}\sum_{x^{n+1}}\mathrm{Pr}\left(X^{n+1}=x^{n+1},f_{n,k}^{*}\left(Y^{n}\right)=b^{k}\right)\cdot {\mathrm{ORD}}_{b^{k},n,n+1}^{s}\left(x^{n+1}\right) \end{array}$$

(A12) $$\begin{array}{cc} \; & \leq 2^{s}\cdot \sum_{b^{k}\in {\left\{0,1\right\}}^{k}}\sum_{x^{n}}\mathrm{Pr}\left(X^{n}=x^{n},f_{n,k}^{*}\left(Y^{n}\right)=b^{k}\right)\cdot {\mathrm{ORD}}_{b^{k},n,n}^{s}\left(x^{n}\right) \end{array}$$

(A13) $$\begin{array}{cc} \; & =2^{s}\cdot G_{s}\left(X^{n}|f_{n,k}^{*}\left(Y^{n}\right)\right). \end{array}$$

Hence,

(A14) $$\gamma_{s,k}^{\left(n+1\right)}\left(\delta \right)\leq \ell_{n}^{-1}\cdot \gamma_{s,k}^{\left(n\right)}\left(\delta \right).$$

To continue, we further analyze $\ell_{n}$. The summation in the numerator of Equation (A7) may be started from from i=2, and so Equations (A31) and (A33) (proof of Lemma 1 below) imply that

(A15) $$\begin{array}{cc} 1 & \geq \ell_{n} \end{array}$$

(A16) $$\begin{array}{cc} \; & \geq \frac{\frac{1}{s+1}\cdot \frac{2^{n(s+1)}-1}{2^{n}-1}}{\frac{1}{s+1}\cdot \frac{{\left(2^{n}+1\right)}^{s+1}-1}{2^{n}}} \end{array}$$

(A17) $$\begin{array}{cc} \; & \geq \frac{2^{n(s+1)}-1}{{\left(2^{n}+1\right)}^{s+1}} \end{array}$$

(A18) $$\begin{array}{cc} \; & ={\left(\frac{2^{n}}{2^{n}+1}\right)}^{s+1}-\frac{1}{2^{n(s+1)}} \end{array}$$

(A19) $$\begin{array}{cc} \; & ={\left(1+\frac{1}{2^{n}}\right)}^{-(s+1)}-\frac{1}{2^{n(s+1)}} \end{array}$$

(A20) $$\begin{array}{cc} \; & =1-\frac{\left(s+1\right)}{2^{n}}+O\left(\frac{1}{2^{2n}}\right)-\frac{1}{2^{n(s+1)}} \end{array}$$

(A21) $$\begin{array}{cc} \; & =1-\frac{\left(s+1\right)}{2^{n}}+O\left(\frac{1}{2^{n\cdot \min\{1+s,2\}}}\right). \end{array}$$

Thus, there exists c,C>0 such that

(A22) $$\begin{array}{cc} \log\prod_{n=1}^{\infty }\ell_{n}^{-1} & =\sum_{n=1}^{\infty }\log\ell_{n}^{-1} \end{array}$$

(A23) $$\begin{array}{cc} \; & \leq -\sum_{n=1}^{\infty }\log\left[1-\frac{c}{2^{n}}\right] \end{array}$$

(A24) $$\begin{array}{cc} \; & \leq C+\sum_{n=1}^{\infty }\frac{c}{2^{n}}+O\left(\frac{1}{2^{2n}}\right) \end{array}$$

(A25) $$\begin{array}{cc} \; & <\infty , \end{array}$$

and consequently,

(A26) $$d_{n}:=\prod_{j=n}^{\infty }\ell_{j}^{-1}\to 1$$

as n→∞. Hence, Equation (A14) implies that

(A27) $$e_{n}:=d_{n}\cdot \gamma_{s}^{\left(n\right)}\left(\delta \right)$$

is a non-increasing sequence which is bounded below by 0 and, thus, has a limit. Since $d_{n}\to 1$ as n→∞, $\gamma_{s}^{\left(n\right)}\left(\delta \right)$ also has a limit. We finally show the reverse ordering property for k=1. The guessing order given that $f(Y^{n})=1$ is determined by ordering

(A28) $$\mathrm{Pr}\left(X^{n}=x^{n}|f\left(Y^{n}\right)=1\right)=\frac{\mathrm{Pr}\left(X^{n}=x^{n}\right)\cdot \mathrm{Pr}\left(f\left(Y^{n}\right)=1|X^{n}=x^{n}\right)}{\mathrm{Pr}(f\left(Y^{n}\right)=1)},$$

or equivalently, by ordering $\mathrm{Pr}(f\left(Y^{n}\right)=1|X^{n}=x^{n})$. It then follows that the order, given that $f(Y^{n})=0$, is reversed compared to the order given that $f(Y^{n})=1$ since

(A29) $$\mathrm{Pr}\left(f\left(Y^{n}\right)=0|X^{n}=x^{n}\right)+\mathrm{Pr}\left(f\left(Y^{n}\right)=1|X^{n}=x^{n}\right)=1.$$

□

**Proof** **of** **Lemma** **1.** The monotonicity of $i^{s}$ and standard bounds on sums using integrals lead to the bounds

(A30) $$\begin{array}{cc} K_{s}\left(a,b\right) & \leq \int_{a+1}^{b+1}\frac{t^{s}}{b-a}\cdot dt \end{array}$$

(A31) $$\begin{array}{cc} \; & =\frac{1}{s+1}\cdot \frac{{\left(b+1\right)}^{s+1}-{\left(a+1\right)}^{s+1}}{b-a} \end{array}$$

and

(A32) $$\begin{array}{cc} K_{s}\left(a,b\right) & \geq \int_{a}^{b}\frac{t^{s}}{b-a}\cdot dt \end{array}$$

(A33) $$\begin{array}{cc} \; & =\frac{1}{s+1}\cdot \frac{b^{s+1}-a^{s+1}}{b-a}. \end{array}$$

The ratio between the upper and lower bound is

(A34) $$\kappa_{s}\left(a,b\right):=\frac{{\left(b+1\right)}^{s+1}-{\left(a+1\right)}^{s+1}}{b^{s+1}-a^{s+1}}$$

which satisfies $\kappa_{s}\left(a_{n},b_{n}\right)\to 1$ given the premise of the lemma. □

**Proof** **of** **Equation** **(61).** Denote by $f_{n}^{*}$ a function which achieves the minimal guessing ratio in Equation (5). Then, it holds that $G_{s}\left(X^{n}|f^{*}\left(Y^{n}\right)=b^{k}\right)$ is a monotonic non-increasing function of *n*. To see this, suppose that $f_{n+1}^{*}$ is an optimal function for n+1. This function $f_{n+1}^{*}$ can be used for guessing $X^{n}$ on the basis of *k* bit of help computed from $Y^{n}$ as follows: Given $Y^{n}$, the helper randomly generates $Y_{n+1}∼P_{Y|X}\left(\cdot |0\right)$, computes $b^{k}=f_{n+1}^{*}\left(Y^{n+1}\right)$, and send these bits to the guesser. The guesser of $X^{n}$ then uses the bits $b^{k}$ to guess $X^{n}$, and the resulting conditional guessing moment is $G_{s}\left(X^{n+1}|f_{n+1}^{*}\left(Y^{n+1}\right)=b^{k},X_{n+1}=0\right)$, which is less than $G_{s}\left(X^{n+1}|f_{n+1}^{*}\left(Y^{n+1}\right)=b^{k}\right)$ since conditioning reduces guessing moments. Thus, the optimal function $f_{n}^{*}$ can only achieve lower guessing moments, which implies the desired monotonicity property. For brevity, we henceforth simply write the optimal function as *f* (with dimension and optimality being implicit). Define the set

(A35) $$B_{k}:=\left\{b^{k}\in {\left\{0,1\right\}}^{k}:\underset{n}{\sup}G_{s}\left(X^{n}|f\left(Y^{n}\right)=b^{k}\right)=\infty \right\},$$

to wit, the set of *k*-tuples such that the conditional guessing moment grows without bound when conditioned on that *k*-tuple. By the law of total expectation

$$\begin{array}{cc} G_{s}\left(X^{n}|f\left(Y^{n}\right)\right) & =\sum_{b^{k}\in B_{k}}\mathrm{Pr}\left(f\left(Y^{n}\right)=b^{k}\right)\cdot G_{s}\left(X^{n}|f\left(Y^{n}\right)=b^{k}\right) \end{array}$$

(A36) =+∑bk∈{0,1}k\BkPr(f(Yn)=bk)·Gs(Xn∣f(Yn)=bk)

(A37) $$\begin{array}{cc} \; & =:G_{n}^{\left(1\right)}+G_{n}^{\left(2\right)}. \end{array}$$

So, since $G_{s}\left(X^{n}|f\left(Y^{n}\right)\right)$ grows without bound as a function of *n*, it must hold that $B_{k}$ is not empty and that there exists $\ell_{n}$ such that $G_{s}\left(X^{n}|f\left(Y^{n}\right)\right)=\ell_{n}G_{n}^{\left(1\right)}$, where $\ell_{n}\to 1$ as n→∞. Let η>0 be given. The monotonicity property previously established and Equation (60) imply that there exists $n_{0}\left(\eta \right)$ such that for all $n\geq n_{0}\left(\eta \right)$ both

(A38) $$G_{s}\left(X^{n}|f\left(Y^{n}\right)=b^{k}\right)\geq \left(1-\eta \right)\cdot \Psi_{s}\cdot 2^{sH(X^{n}|f\left(Y^{n}\right)=b^{k})}$$

and

(A39) $$\Psi_{s}\cdot 2^{sH(X^{n}|f\left(Y^{n}\right)=b^{k})}\geq \left(1-\eta \right)\cdot G_{s}\left(X^{n}|f\left(Y^{n}\right)=b^{k}\right)$$

hold for any $b^{k}\in B_{k}$. Thus, also

(A40) $$G_{s}\left(X^{n}|f\left(Y^{n}\right)\right)\geq \ell_{n}\left(1-\eta \right)\sum_{b^{k}\in B_{k}}\mathrm{Pr}\left(f\left(Y^{n}\right)=b^{k}\right)\cdot \Psi_{s}\cdot 2^{sH(X^{n}|f\left(Y^{n}\right)=b^{k})}$$

and

(A41) $$\sum_{b^{k}\in B_{k}}\mathrm{Pr}\left(f\left(Y^{n}\right)=b^{k}\right)\cdot \Psi_{s}\cdot 2^{sH(X^{n}|f\left(Y^{n}\right)=b^{k})}\geq \sum_{b^{k}\in B_{k}}\mathrm{Pr}\left(f\left(Y^{n}\right)=b^{k}\right)\left(1-\eta \right)\cdot G_{s}\left(X^{n}|f\left(Y^{n}\right)=b^{k}\right)$$

hold, and the last equation implies that the term on its left-hand side is unbounded. Moreover, Equation (60) and the sentence that follows it both imply that, if $G_{s}\left(X^{n}|f\left(Y^{n}\right)=b^{k}\right)$ is bounded, then $H(X^{n}|f\left(Y^{n}\right)=b^{k})$ is bounded too. Thus, there exists $k_{n}$ which satisfies $k_{n}\to 1$ as n→∞ such that

(A42) $$\sum_{b^{k}\in B_{k}}\mathrm{Pr}\left(f\left(Y^{n}\right)=b^{k}\right)\cdot \Psi_{s}\cdot 2^{sH(X^{n}|f\left(Y^{n}\right)=b^{k})}=k_{n}\cdot \sum_{b^{k}\in {\left\{0,1\right\}}^{n}}\mathrm{Pr}\left(f\left(Y^{n}\right)=b^{k}\right)\cdot \Psi_{s}\cdot 2^{sH(X^{n}|f\left(Y^{n}\right)=b^{k})}.$$

Combining Equation (A40) with the last equation and noting that η>0 is arbitrary completes the proof. □

**Proof** **of** **Theorem** **6.** The proof follows the same lines as the proof of Theorem 3 up to Equation (62), yielding

(A43) $$G_{s}\left(X^{n}|f\left(Y^{n}\right)\right)\geq k_{n}\cdot \Psi_{s}\cdot 2^{s\left[n-I(X^{n};f\left(Y^{n}\right))\right]}.$$

Now, let $W^{\left(n\right)}$ be such that $X^{n}-Y^{n}-W^{\left(n\right)}$ forms a Markov chain. Then,

(A44) $$\begin{array}{cc} \underset{f:Y^{n}\to \left\{0,1\right\}}{\sup}\frac{I(X^{n};f\left(Y^{n}\right))}{I(Y^{n};f\left(Y^{n}\right))} & \leq \underset{P_{W^{\left(n\right)}|Y^{n}}}{\sup}\frac{I(X^{n};W^{\left(n\right)})}{I(Y^{n};W^{\left(n\right)})} \end{array}$$

(A45) $$\begin{array}{cc} \; & =\eta (P_{Y^{n}},P_{X^{n}|Y^{n}}) \end{array}$$

(A46) $$\begin{array}{cc} \; & =\eta (P_{Y},P_{X|Y}), \end{array}$$

where Equation (A46) follows since the SDPI constant tensorizes (see Reference [40] for an argument obtained by relating the SDPI constant to the hypercontractivity parameter or its extended version, Reference ([40], p. 5), for a direct proof). Thus, for all *f*,

(A47) $$\begin{array}{cc} I(X^{n};f\left(Y^{n}\right)) & \leq \eta \left(P_{Y},P_{X|Y}\right)\cdot I\left(Y^{n};f\left(Y^{n}\right)\right) \end{array}$$

(A48) $$\begin{array}{cc} \; & \leq \eta \left(P_{Y},P_{X|Y}\right)\cdot H\left(f\left(Y^{n}\right)\right) \end{array}$$

(A49) $$\begin{array}{cc} \; & \leq \eta (P_{Y},P_{X|Y})\cdot k. \end{array}$$

Inserting Equation (A49) into Equation (A43) yields

(A50) $$G_{s}\left(X^{n}|f\left(Y^{n}\right)\right)\geq k_{n}\cdot \Psi_{s}\cdot 2^{s\left[n-k\cdot \eta (P_{Y},P_{X|Y})\right]},$$

and substituting this in the definition of the guessing ratio of Equation (5) completes the proof. □

**Proof** **of** **Equation** **(100).** Let us evaluate the posterior probability conditioned on $\text{G-Dict}(Y^{n})=0$. Since G-Dict is balanced, Bayes law implies that

$$\begin{array}{cc} \; & \mathrm{Pr}(X^{n}=x^{n}|\text{G-Dict}\left(Y^{n}\right)=0) \end{array}$$

(A51) $$\begin{array}{cc} \; & =2^{-(n-1)}\cdot \mathrm{Pr}\left(\text{G-Dict}\left(Y^{n}\right)=0|X^{n}=x^{n}\right) \end{array}$$

(A52) $$\begin{array}{cc} \; & =2^{-(n-1)}\cdot \sum_{i=1}^{n+1}\mathrm{Pr}\left(k\left(y^{n}\right)=i|X^{n}=x^{n}\right)\cdot \mathrm{Pr}\left(\text{G-Dict}\left(Y^{n}\right)=0|X^{n}=x^{n},k\left(y^{n}\right)=i\right) \end{array}$$

(A53) $$\begin{array}{cc} \; & =2^{-(n-1)}\cdot \left\{\sum_{i=1}^{n}\left(1-\epsilon \right)\epsilon^{i-1}\cdot 𝟙\left\{x_{i}=0\right\}+\frac{1}{2}\epsilon^{n}\right\}. \end{array}$$

This immediately leads to the guessing rule in Equation (100). From Proposition 1, the guessing rule for $\text{G-Dict}(Y^{n})=1$ is on reverse order. □

**Proof** **of** **Proposition** **3.** We denote the lexicographic order by ${\mathrm{ORD}}_{\mathrm{lex}}$. Assume that $\text{G-Dict}(Y^{n})=0$ and that ${\mathrm{ORD}}_{\mathrm{lex}}\left(x^{n}\right)\leq {\mathrm{ORD}}_{\mathrm{lex}}\left(z^{n}\right)$. Then, there exists j∈[n] such that $x^{j-1}=z^{j-1}$ (where $x^{0}$ is the empty string) and $x_{j}=0<z_{j}=1$. Then,

$$\begin{array}{cc} \; & \mathrm{Pr}\left(X^{n}=x^{n}|\text{G-Dict}\left(Y^{n}\right)=0\right)-\mathrm{Pr}\left(X^{n}=z^{n}|\text{G-Dict}\left(Y^{n}\right)=0\right) \end{array}$$

(A54) $$\begin{array}{cc} \; & =\epsilon^{j-1}+\sum_{i=j+1}^{n}\epsilon^{i-1}\cdot \left(z_{i}-x_{i}\right) \end{array}$$

(A55) $$\begin{array}{cc} \; & \geq \epsilon^{j-1}-\sum_{i=j+1}^{n}\epsilon^{i-1} \end{array}$$

(A56) $$\begin{array}{cc} \; & =\frac{\epsilon^{j-1}}{1-\epsilon }\left(1-2\epsilon +\epsilon^{n-j+1}\right) \end{array}$$

(A57) $$\begin{array}{cc} \; & \geq 0. \end{array}$$

This proves the first statement of the proposition. Now, let ${\mathrm{ORD}}_{0}$ (${\mathrm{ORD}}_{1}$) be the guessing order given that the received bit is 0 (resp. 1), and let $\left\{f_{S}\right\}$ be the Boolean functions (which are not necessarily optimal). Then, from Equations (97) and (95)

$$\begin{array}{cc} \; & G_{1}\left(X^{n}|f\left(Y^{n}\right)\right) \end{array}$$

(A58) $$\begin{array}{cc} \; & =\sum_{x^{n}}\mathrm{Pr}\left(X^{n}=x^{n},f\left(Y^{n}\right)=0\right)\cdot {\mathrm{ORD}}_{0}\left(x^{n}\right)+\mathrm{Pr}\left(X^{n}=x^{n},f\left(Y^{n}\right)=1\right)\cdot {\mathrm{ORD}}_{1}\left(x^{n}\right) \end{array}$$

(A59) $$\begin{array}{cc} \; & =2^{-n}\cdot \sum_{S\subseteq [n]}\mathrm{Pr}\left(S\right)\sum_{x^{n}}\left[\left(1-f_{S}\left(x^{n}\right)\right)\cdot {\mathrm{ORD}}_{0}\left(x^{n}\right)+f_{S}\left(x^{n}\right)\cdot {\mathrm{ORD}}_{1}\left(x^{n}\right)\right] \end{array}$$

(A60) $$\begin{array}{cc} \; & =2^{-n}\cdot \sum_{S\subseteq [n]}\mathrm{Pr}\left(S\right)\sum_{x^{S}}\left[\left(1-f_{S}\left(x^{n}\right)\right)\cdot {\mathrm{PORD}}_{0}(x^{S}\left||S\right)+f_{S}\left(x^{n}\right)\cdot {\mathrm{PORD}}_{1}(x^{S}\left||S\right)\right] \end{array}$$

(A61) $$\begin{array}{cc} \; & \geq 2^{-n}\cdot \sum_{S\subseteq [n]}\mathrm{Pr}\left(S\right)\sum_{x^{n}}\min\left\{{\mathrm{PORD}}_{0}(x^{S}||S),{\mathrm{PORD}}_{1}(x^{S}\left||S\right)\right\}, \end{array}$$

where for b∈{0,1}, the *projected orders* are defined as

(A62) $${\mathrm{PORD}}_{b}(x^{S}||S):=\sum_{x^{\left(S^{c}\right)}}{\mathrm{ORD}}_{b}\left(x^{n}\right).$$

It is easy to verify that, if ${\mathrm{ORD}}_{0}$ (${\mathrm{ORD}}_{1}$) is the lexicographic (resp. revered lexicographic) order, then the greedy dictator achieves Equation (A61) with equality due to the following simple property: If ${\mathrm{ORD}}_{\mathrm{lex}}\left(x^{n}\right)<{\mathrm{ORD}}_{\mathrm{lex}}\left(z^{n}\right)$, then

(A63) $$\sum_{x^{\left(S^{c}\right)}}{\mathrm{ORD}}_{\mathrm{lex}}\left(x^{n}\right)\leq \sum_{x^{\left(S^{c}\right)}}{\mathrm{ORD}}_{\mathrm{lex}}\left(z^{n}\right)$$

for all S∈[n]. This can be proved by induction over *n*. For n=1, the claim is easily asserted. Suppose it holds for n−1, let us verify it for *n*. If 1∈S, then whenever ${\mathrm{ORD}}_{\mathrm{lex}}\left(x^{n}\right)<{\mathrm{ORD}}_{\mathrm{lex}}\left(z^{n}\right)$

(A64) $$\begin{array}{cc} \sum_{x^{\left(S^{c}\right)}}{\mathrm{ORD}}_{\mathrm{lex}}\left(x^{n}\right) & =\sum_{x^{\left(S^{c}\right)}}{\mathrm{ORD}}_{\mathrm{lex}}\left(x_{1},x_{2}^{n}\right) \end{array}$$

(A65) $$\begin{array}{cc} \; & =x_{1}\cdot 2^{n-1}+\sum_{x^{\left(S^{c}\right)}}{\mathrm{ORD}}_{\mathrm{lex}}\left(x_{2}^{n}\right) \end{array}$$

(A66) $$\begin{array}{cc} \; & \leq z_{1}\cdot 2^{n-1}+\sum_{z^{\left(S^{c}\right)}}{\mathrm{ORD}}_{\mathrm{lex}}\left(z_{2}^{n}\right) \end{array}$$

(A67) $$\begin{array}{cc} \; & =\sum_{z^{\left(S^{c}\right)}}{\mathrm{ORD}}_{\mathrm{lex}}\left(z^{n}\right) \end{array}$$

where the inequality follows from the induction assumption and since $x_{1}\leq z_{1}$. If 1∉S then, similarly,

(A68) $$\begin{array}{cc} \sum_{x^{\left(S^{c}\right)}}{\mathrm{ORD}}_{\mathrm{lex}}\left(x^{n}\right) & =\sum_{x^{\left(S^{c}\backslash \left\{1\right\}\right)}}\left[2^{n-1}+2\cdot {\mathrm{ORD}}_{\mathrm{lex}}\left(x_{2}^{n}\right)\right] \end{array}$$

(A69) $$\begin{array}{cc} \; & \leq \sum_{z^{\left(S^{c}\backslash \left\{1\right\}\right)}}\left[2^{n-1}+2\cdot {\mathrm{ORD}}_{\mathrm{lex}}\left(z_{2}^{n}\right)\right] \end{array}$$

(A70) $$\begin{array}{cc} \; & =\sum_{z^{\left(S^{c}\right)}}{\mathrm{ORD}}_{\mathrm{lex}}\left(z^{n}\right). \end{array}$$

□

**Proof** **of** **Theorem** **7.** We denote the lexicographic order by ${\mathrm{ORD}}_{\mathrm{lex}}$. Then,

(A71) $$\begin{array}{cc} G_{1}\left(X^{n}|\text{G-Dict}\left(Y^{n}\right)\right) & =G_{1}\left(X^{n}|\text{G-Dict}\left(Y^{n}\right)=0\right) \end{array}$$

(A72) $$\begin{array}{cc} \; & \leq \sum_{x^{n}}\mathrm{Pr}\left(X^{n}=x^{n}|\text{G-Dict}\left(Y^{n}\right)=0\right)\cdot {\mathrm{ORD}}_{\mathrm{lex}}\left(x^{n}\right) \end{array}$$

(A73) $$\begin{array}{cc} \; & =2^{-(n-1)}\cdot \sum_{x^{n}}\sum_{i=1}^{n}\left(1-\epsilon \right)\epsilon^{i-1}\cdot 𝟙\left\{x_{i}=0\right\}\cdot {\mathrm{ORD}}_{\mathrm{lex}}\left(x^{n}\right)+\epsilon^{n}K_{1}\left(2^{n}\right) \end{array}$$

(A74) $$\begin{array}{cc} \; & =2^{-(n-1)}\cdot \left(1-\epsilon \right)\sum_{i=1}^{n}\epsilon^{i-1}\cdot \sum_{x^{n}}𝟙\left\{x_{i}=0\right\}\cdot {\mathrm{ORD}}_{\mathrm{lex}}\left(x^{n}\right)+\epsilon^{n}K_{1}\left(2^{n}\right) \end{array}$$

(A75) $$\begin{array}{cc} \; & =\left(1-\epsilon \right)J_{n}+\epsilon^{n}K_{1}\left(2^{n}\right), \end{array}$$

where $J_{1}:=\frac{1}{2}$ and for n≥2

(A76) $$\begin{array}{cc} J_{n} & :=2^{-(n-1)}\cdot \sum_{i=1}^{n}\epsilon^{i-1}\cdot \sum_{x^{n}}𝟙\left\{x_{i}=0\right\}\cdot {\mathrm{ORD}}_{\mathrm{lex}}\left(x^{n}\right) \end{array}$$

(A77) $$\begin{array}{cc} \; & =2^{-(n-1)}\sum_{x^{n}}𝟙\left\{x_{i}=0\right\}\cdot {\mathrm{ORD}}_{\mathrm{lex}}\left(x^{n}\right)+2^{-(n-1)}\sum_{i=2}^{n}\epsilon^{i-1}\cdot \sum_{x^{n}}𝟙\left\{x_{i}=0\right\}\cdot {\mathrm{ORD}}_{\mathrm{lex}}\left(x^{n}\right)\\ \; & =K_{1}\left(2^{n-1}\right) \end{array}$$

(A78) =+2−(n−1)∑i=2nϵi−1·∑x2n𝟙x1=0,xi=0·ORDlex(0,x2n)+𝟙x1=1,xi=0·ORDlex(1,x2n)=K1(2n−1)+2−(n−1)ϵ∑i=1n−1ϵi−1·∑xn−1𝟙xi=0ORDlex(xn−1)

(A79) =+2−(n−1)ϵ∑i=1n−1ϵi−1·∑xn−1𝟙xi=02n−1+ORDlex(xn−1)

(A80) $$\begin{array}{cc} \; & =K_{1}\left(2^{n-1}\right)+\epsilon J_{n-1}+\sum_{i=1}^{n-1}\epsilon^{i}\cdot \sum_{x^{n-1}}𝟙\left\{x_{i}=0\right\} \end{array}$$

(A81) $$\begin{array}{cc} \; & =K_{1}\left(2^{n-1}\right)+\epsilon J_{n-1}+2^{n-2}\cdot \frac{\epsilon -\epsilon^{n}}{1-\epsilon }. \end{array}$$

So,

(A82) $$\begin{array}{cc} J_{n} & =K_{1}\left(2^{n-1}\right)+\epsilon \left[K_{1}\left(2^{n-2}\right)+\epsilon J_{n-2}+2^{n-3}\cdot \frac{\epsilon -\epsilon^{n-1}}{1-\epsilon }\right]+2^{n-2}\cdot \frac{\epsilon -\epsilon^{n}}{1-\epsilon } \end{array}$$

(A83) $$\begin{array}{cc} \; & =K_{1}\left(2^{n-1}\right)+\epsilon K_{1}\left(2^{n-2}\right)+\epsilon^{2}J_{n-2}+2^{n-3}\cdot \frac{\epsilon^{2}-\epsilon^{n}}{1-\epsilon }+2^{n-2}\cdot \frac{\epsilon -\epsilon^{n}}{1-\epsilon } \end{array}$$

(A84) $$\begin{array}{cc} \; & =\sum_{i=1}^{n}\epsilon^{i-1}K_{1}\left(2^{n-i}\right)+\frac{1}{1-\epsilon }\sum_{i=1}^{n}2^{i-2}\cdot \left(\epsilon^{n-i+1}-\epsilon^{n}\right). \end{array}$$

Hence,

(A85) $$G_{1}\left(X^{n}|\text{G-Dict}\left(Y^{n}\right)\right)\leq \left(1-\epsilon \right)\sum_{i=1}^{n}\epsilon^{i-1}K_{1}\left(2^{n-i}\right)+\sum_{i=1}^{n}2^{i-2}\cdot \left(\epsilon^{n-i+1}-\epsilon^{n}\right)+\epsilon^{n}K_{1}\left(2^{n}\right).$$

Noting that $K_{1}\left(M\right)=\frac{M+1}{2}$, we get

$$\begin{array}{cc} \; & G_{1}\left(X^{n}|\text{G-Dict}\left(Y^{n}\right)\right) \end{array}$$

(A86) $$\begin{array}{cc} \; & \leq 2^{n-1}\frac{\left(1-\epsilon \right)}{\epsilon }\sum_{i=1}^{n}{\left(\frac{\epsilon }{2}\right)}^{i}+\frac{\left(1-\epsilon \right)\left(1-\epsilon^{n}\right)}{2\epsilon }+\frac{1}{4}\sum_{i=1}^{n}{\left(\frac{2}{\epsilon }\right)}^{i}\cdot \epsilon^{n+1}-\frac{1}{2}\left(2^{n}-1\right)\epsilon^{n}+2^{n-1}\epsilon^{n}+\frac{\epsilon^{n}}{2} \end{array}$$

(A87) $$\begin{array}{cc} \; & =\frac{1}{2-\epsilon }\left(2^{n-1}+\frac{\epsilon^{n}}{2}\left(1-\epsilon \right)\right)+\frac{\left(1-\epsilon \right)\left(1-\epsilon^{n}\right)}{2\epsilon } \end{array}$$

(A88) $$\begin{array}{cc} \; & ≐\frac{2^{n-1}}{2-\epsilon }. \end{array}$$

□

## References

[1] Arikan E. (1996). An inequality on guessing and its application to sequential decoding. IEEE Trans. Inf. Theory.

[2] Arikan E., Merhav N. (1998). Guessing subject to distortion. IEEE Trans. Inf. Theory.

[3] Merhav N., Arikan E. (1999). The Shannon cipher system with a guessing wiretapper. IEEE Trans. Inf. Theory.

[4] Hayashi Y., Yamamoto H. (2008). Coding theorems for the Shannon cipher system with a guessing wiretapper and correlated source outputs. IEEE Trans. Inf. Theory.

[5] Hanawal M.K., Sundaresan R. (2011). The Shannon cipher system with a guessing wiretapper: General sources. IEEE Trans. Inf. Theory.

[6] Christiansen M.M., Duffy K.R., du Pin Calmon F., Médard M. (2015). Multi-user guesswork and brute force security. IEEE Trans. Inf. Theory.

[7] Yona Y., Diggavi S. The effect of bias on the guesswork of hash functions. Proceedings of the 2017 IEEE International Symposium on Information Theory (ISIT).

[8] Massey J.L. Guessing and entropy. Proceedings of the 1994 IEEE International Symposium on Information Theory.

[9] Arikan E. Large deviations of probability rank. Proceedings of the 2000 IEEE International Symposium on Information Theory.

[10] Christiansen M.M., Duffy K.R. (2012). Guesswork, large deviations, and Shannon entropy. IEEE Trans. Inf. Theory.

[11] Pfister C.E., Sullivan W.G. (2004). Rényi entropy, guesswork moments, and large deviations. IEEE Trans. Inf. Theory.

[12] Hanawal M.K., Sundaresan R. (2011). Guessing revisited: A large deviations approach. IEEE Trans. Inf. Theory.

[13] Sundaresan R. (2007). Guessing under source uncertainty. IEEE Trans. Inf. Theory.

[14] Serdar B. (1997). Comments on “An inequality on guessing and its application to sequential decoding”. IEEE Trans. Inf. Theory.

[15] Sason I., Verdú S. (2018). Improved bounds on lossless source coding and guessing moments via Rényi measures. IEEE Trans. Inf. Theory.

[16] Sason I. (2018). Tight bounds on the Rényi entropy via majorization with applications to guessing and compression. Entropy.

[17] Wyner A. (1973). A theorem on the entropy of certain binary sequences and applications—II. IEEE Trans. Inf. Theory.

[18] Ahlswede R., Körner J. (1975). Source coding with side information and a converse for degraded broadcast channels. IEEE Trans. Inf. Theory.

[19] Graczyk R., Lapidoth A. Variations on the guessing problem. Proceedings of the 2018 IEEE International Symposium on Information Theory.

[20] Graczyk R. (2017). Guessing with a Helper. Master’s Thesis.

[21] O’Donnell R. (2014). Analysis of Boolean Functions.

[22] Courtade T.A., Kumar G.R. (2014). Which Boolean functions maximize mutual information on noisy inputs?. IEEE Trans. Inf. Theory.

[23] Ordentlich O., Shayevitz O., Weinstein O. An improved upper bound for the most informative Boolean function conjecture. Proceedings of the 2016 IEEE International Symposium on Information Theory.

[24] Samorodnitsky A. (2016). On the entropy of a noisy function. IEEE Trans. Inf. Theory.

[25] Kindler G., O’Donnell R., Witmer D. Continuous Analogues of the most Informative Function Problem. http://arxiv.org/pdf/1506.03167.pdf.

[26] Li J., Médard M. Boolean functions: Noise stability, non-interactive correlation, and mutual information. Proceedings of the 2018 IEEE International Symposium on Information Theory.

[27] Chandar V., Tchamkerten A. Most informative quantization functions. Presented at the 2014 Information Theory and Applications Workshop.

[28] Weinberger N., Shayevitz O. (2017). On the optimal Boolean function for prediction under quadratic Loss. IEEE Trans. Inf. Theory.

[29] Burin A., Shayevitz O. (2018). Reducing guesswork via an unreliable oracle. IEEE Trans. Inf. Theory.

[30] Ardimanov N., Shayevitz O., Tamo I. Minimum Guesswork with an Unreliable Oracle. Proceedings of the 2018 IEEE International Symposium Information Theory.

[31] Feller W. (1971). An Introduction to Probability Theory and Its Applications.

[32] Cover T.M., Thomas J.A. (2006). Elements of Information Theory.

[33] Wainwright M.J., Jordan M.I. (2008). Graphical models, exponential families, and variational inference. Found. Trends® Mach. Learn..

[34] Boyd S.P., Vandenberghe L. (2004). Convex Optimization.

[35] Nadarajah S. (2005). A generalized normal distribution. J. Appl. Stat..

[36] Wyner A., Ziv J. (1973). A theorem on the entropy of certain binary sequences and applications—I. IEEE Trans. Inf. Theory.

[37] Erkip E., Cover T.M. (1998). The efficiency of investment information. IEEE Trans. Inf. Theory.

[38] Ahlswede R., Gács P. (1976). Spreading of sets in product spaces and hypercontraction of the Markov operator. Ann. Probab..

[39] Raginsky M. (2016). Strong data processing inequalities and Φ–Sobolev inequalities for discrete channels. IEEE Trans. Inf. Theory.

[40] Anantharam V., Gohari A., Kamath S., Nair C. On hypercontractivity and a data processing inequality. Proceedings of the 2014 IEEE International Symposium on Information Theory.

